# A multidisciplinary approach disclosing unexplored Aflatoxin B1 roles in severe impairment of vitamin D mechanisms of action

**DOI:** 10.1007/s10565-022-09752-y

**Published:** 2022-09-06

**Authors:** Marco Persico, Raffaele Sessa, Elena Cesaro, Irene Dini, Paola Costanzo, Alberto Ritieni, Caterina Fattorusso, Michela Grosso

**Affiliations:** 1 Department of Pharmacy, University of Naples Federico II, Via Domenico Montesano, Naples, Italy; 2 Department of Molecular Medicine and Medical Biotechnology, University of Naples Federico II, Via Sergio Pansini, Naples, Italy; 3 Staff of UNESCO Chair On Health Education and Sustainable Development, University of Naples Federico II, Naples, Italy

**Keywords:** Aflatoxin B1, Vitamin D receptor, Retinoid receptors, Docking studies, Gene expression, Transcriptional regulation

## Abstract

Aflatoxin B1 (AFB1), produced by fungi of the genus *Aspergillus*, is the most toxic and carcinogenic mycotoxin among the classes of aflatoxins. Previous research showed that AFB1 affects vitamin D receptor (VDR) expression. In the present study, integrated computational and experimental studies were carried out to investigate how AFB1 can interfere with Vitamin D signalling. A competitive antagonism of AFB1 toward RXRα and VDR was hypothesized by comparing the docked complex of AFB1/RXRα and AFB1/VDR ligand-binding domain (LBD) with the X-ray structures of RXRα and VDR bound to known ligands. Accordingly, we demonstrated that AFB1 can affect vitamin D-mediated transcriptional activation of VDR by impairing the formation of protein complexes containing both VDR-RXRα and RXRα/RAR and affecting the subcellular localization of VDR and RXRα. As a whole, our data indicate that AFB1 can interfere with different molecular pathways triggered by vitamin D with an antagonistic mechanism of action.

**Supplementary Information:**

The online version contains supplementary material available at 10.1007/s10565-022-09752-y.

## Introduction

**Fig. 1:**
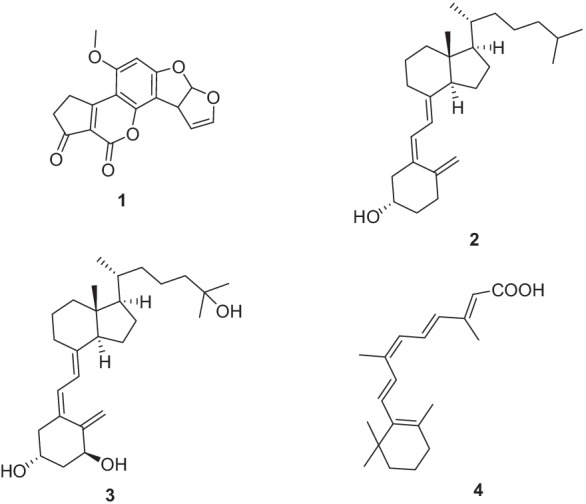
Structure of Aflatoxin B1 (**1**), vitamin D_3_ (**2**),1,25-dihydroxyvitamin D_3_ (**3**), and 9-*cis*-Retinoic Acid (**4**)

Mycotoxins are secondary metabolites produced by filamentous fungi universally present in foods and feeds. Mycotoxin contamination can occur directly in the field or during processing and storage procedures when environmental conditions are favorable to fungal colonization and growth, such as warm, humid climates or temperate areas during drought. A large proportion of the world population is thus expected to be chronically exposed to mycotoxins, especially in developing and under-developed countries with exposure risks to mycotoxin ever growing because of global climate changes (Sabir et al. 2021; Gruber-Dorninger et al. 2019; Ezekiel et al. 2018). In the last decades, a large body of literature has accumulated providing evidence of toxigenic, mutagenic, and immunosuppressive effects of mycotoxin exposure that, along with data on the natural occurrence of these contaminants, contribute to the evaluation of the safety level of the entire food chain. This is of great relevance since mycotoxin prevalence in food crops is estimated up to 60–80% and poses a severe risk to human health, even at low-dose chronic exposure (Eskola et al. 2020). Consequently, mycotoxin contamination represents a global relevant risk to human and animal health. Aflatoxins are mycotoxins produced by specific fungi of the genus *Aspergillus* and are considered worldwide unavoidable food and feed contaminants (Benkerroum 2020; Ismail et al. 2021; Caceres et al. 2020). Among this group of mycotoxins, Aflatoxin B1 (AFB1) (**1**, Fig. 1) is widely recognized as the most toxic and carcinogenic compound that contaminates a wide variety of products usually used in the human diet for which the highest levels of food safety have been set (As Low As Reasonably Achievable, ALARA) (Marchese et al. 2018). Since its discovery, intensive research has been carried out to investigate the mechanisms of AFB1 toxicity. AFB1 is metabolized in the liver by microsomal enzymes to hydroxyl, hydrate, demethyl, and epoxidate derivatives (Dhanasekaran et al. 2011), and excreted in the urine, feces, and milk of lactating animals (Dhanasekaran et al. 2011; Heimburger 2014; Adejumo et al. 2013). Among these derivatives, the toxicological effects of AFB1-8, 9-epoxide (AFBO) have been well established. AFBO can directly interact with DNA to generate highly mutagenic AFB1-DNA adducts responsible for genomic instability and increased cancer risk (Engin and Engin 2019). However, other toxicological mechanisms so far ascribed to AFB1 remain to be elucidated. Recent epidemiology studies showed that aflatoxin exposure through dietary sources in early life contributes to malnutrition and growth retardation in children from developing countries especially in populations of rural areas that are mostly impacted by drought and food insecurity (Wangia-Dixon et al. 2020; da Silva et al. 2021; Dini and Laneri, 2021). In this context, aflatoxin exposure was also found to be inversely associated with insulin like growth factor (IGF1) and IGF binding protein 3 (IGFBP3) levels in Kenyan schoolchildren, thus supporting the hypothesis that the IGF growth axis plays a role in aflatoxin-associated child growth impairment (Castelino et al. 2015). Furthermore, the possible effects of AFB1 as an endocrine disruptor on pituitary gland, thyroid gland and gonads have also been proposed in high-risk worker populations (Beshir et al. 2020; Chen et al. 2019; Wangia et al. 2019). Taken together, these studies demonstrate the importance of aflatoxin exposure especially on children’s health, and more importantly, the need of mechanistic studies to better understand the basis of toxicity of this class of mycotoxins. However, apart from the AFB1-DNA adducts generated by the AFB1-8,9 epoxide metabolite, little is known so far regarding this issue, probably due to the complexity of the molecular mechanisms underlying the patterns of AFB1 toxicity and the intricate risk factors, some of which may be confounding factors. Therefore, despite the intensive work that has been carried out in the last decades since its discovery, the toxicity mechanisms of AFB1 require further research to clarify many essential aspects.

Bearing this in mind and prompted by our previous observations showing AFB1 inhibitory effects on vitamin D receptor (VDR) expression, we addressed our efforts to investigate the molecular mechanism through which AFB1 hinders vitamin D activity. In fact, in 2015 Costanzo et al. had reported the toxic effects of AFB1 towards the vitamin D receptors in osteosarcoma cell line Saos-2 where the expression of vitamin D receptor was significantly down-modulated after exposure to ABF1. This study had also raised the hypothesis that AFB1 could interfere with the actions of vitamin D on calcium-regulated gene expression thus increasing the risk of developing rickets in infant populations chronically exposed to mycotoxins through food chain.

Vitamins D are a group of seven secosteroids fat-soluble hormones ingested in dietary sources or produced in the skin when sun exposure occurs (Dusso et al. 2005). In humans, vitamin D_3_ (also known as cholecalciferol; **2**, Fig. 1) and vitamin D_2_ (ergocalciferol) are essential bioactive molecules of this group of steroidal hormones. 1,25(OH)_2_D_3_ (**3**, Fig. 1) is the active form of vitamin D_3_. It regulates calcium homeostasis, induces differentiation, and inhibits the proliferation of various normal and cancer cells, including osteoclasts, keratinocytes, and monocytes (Holick 2007; Gocek et al. 2007). It is known that the primary regulator of the biological activity of 1,25(OH)_2_D_3_ is the nuclear vitamin D receptor (VDR). VDR is a ligand-dependent transcription factor and a nuclear receptor superfamily member. Like the majority of nuclear receptors, VDR regulates transcription by recognizing and binding specific responsive elements (VDREs) as a nonpermissive heterodimer with retinoid X receptor RXRα (nonpermissive heterodimers are those that can only be activated by the partner’s ligand) (Zhang et al. 2011; Szanto et al. 2004).

RXRs belong to the family of nuclear hormone receptors. The two families of retinoid receptors (RARs and RXRs) consist of three isotypes, α, β, and γ. RARs can be activated by all-trans retinoic acid (ATRA) and 9-*cis* retinoic acid (RA) (**4**, Fig. 1), while RXRs can be activated only by 9-cis RA ( Szanto et al. 2004). These receptors function as ligand-activated transcription factors and mediate the pleiotropic effects of retinoids by activating or repressing the expression of a large array of genes that are critical for cell growth, differentiation and cell death (Xu et al. 2017; Trombetti et al. 2021). Nuclear receptors are composed of three principal domains, a variable N-terminal domain that contains a ligand-independent activation function, a central highly conserved DNA-binding domain (DBD), and a large C-terminal ligand-binding domain (LBD), with a short linker between DBD and LBD (Kakuda et al. 2010; Ghosh et al. 2002). The LBDs of RXRs and VDR share a typical overall structure compared to other nuclear receptors. LBD is a multifunction domain capable of ligand binding, dimerization, and interactions with other partner proteins (including nuclear transporters, co-activators, and co-repressors). The C-terminal helix 12, termed AF-2, controls LBD ability to activate transcription (Kakuda et al. 2010; Sánchez-Martínez et al. 2008). The binding of 1,25(OH)_2_D3 to VDR leads to conformational changes in the receptor, promoting active VDR-RXR heterodimers (Zella et al. 2006). The activated RXR–VDR heterodimer recruits co-regulator complexes in proximity to DNA to remodel chromatin and alter gene transcription in a ligand-dependent manner. Also, 1,25(OH)_2_D_3_ up-regulates the expression of its receptor gene, thus modulating the levels of VDR (Zella et al. 2006). Few previous reports suggest that AFB1 may affect vitamin D function and calcium metabolism. Indeed, exposure to AFB1 in broiler chicks was found to lower serum concentrations of vitamin D and calcium supposedly by impairing renal function and parathyroid metabolism (Glahn et al. 1991; Rushing and Selim 2019). However, a mechanistic explanation of these observations remains lacking (Barac 2019; Cao et al. 2022). Therefore, to clarify this issue, a multidisciplinary approach based on integrated computational and experimental studies was used to investigate the putative mechanisms by which AFB1 can affect vitamin D-mediated VDR expression. Our findings revealed a previously unexplored antagonistic role of AFB1 against Vitamin D activity and highlighted possible molecular interactions between AFB1 and RXRα. Given the impact of retinoid-responsive genes on a wide range of biological processes including development, differentiation, proliferation, and apoptosis, this study provides proof of concept for the relationship between chronic exposure to AFB1 and the onset of relevant clinical manifestations in populations at risk for mycotoxins.

## Results

### Molecular modeling studies

**Fig. 2:**
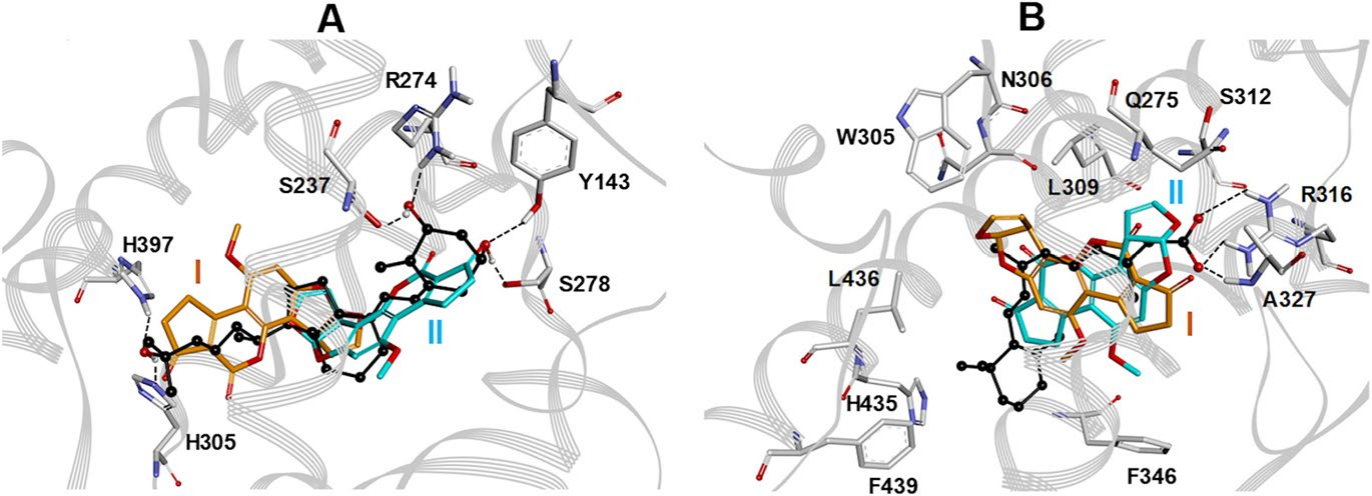
**A**: The two different superimpositions of AFB1 on the vitamin D_3_ (black) in complex with VDR (PDB ID: 1DB1) used to generate the starting binding mode I (orange) and II (cyan). **B**: The two different superimpositions of AFB1 on the 9-cis retinoic acid (black) in complex with RxRα (PDB ID: 1FBY) used to generate the starting binding mode I (orange) and II (cyan)

Molecular modeling studies were performed to investigate the putative interaction of AFB1 with the LBD of VDR and RxRα. The available experimentally determined structures of VDR and RxRα were downloaded from the Brookhaven Protein Data Bank (http://www.rcsb.org) and subjected to a structural and bioinformatics analysis in order to: i) choose the structure to be used as protein starting conformation in docking studies; ii) generate the AFB1 starting binding poses (docking starting complexes); iii) map the experimentally determined ligand–protein interactions and ligand induced protein conformational changes for the interpretation of docking results (for details, see Material and Methods). In the case of hVDR LBD, the full-length structure with the highest resolution (1.45 Ǻ) was selected (PDB ID 3A40). On the other hand, the full length structure of hRXRα was not available and a molecular model has been built by combining two very similar high-resolution structures (RMSD on Cα atoms = 1.14 Ǻ), such as 2P1U (2.20 Ǻ) and 1FM6 (2.10 Ǻ). Dynamic docking studies were performed using a Monte Carlo/Simulated Annealing (SA) docking methodology (Affinity, SA-Docking; Insight 2005, Accelrys, San Diego, CA) (Senderowitz et al. 1995) and the Cell Multipole method for non-bond interactions (Ding et al.^1992^). The binding domain area was defined as a flexible subset around the ligand, including all residues and water molecules having at least one atom within a 10 Å radius from any given ligand atom. Although during the docking simulations, all atoms of the binding domain area, including the ligand, were left free to move, nevertheless, to improve the reliability of the docking results further, the variance of the starting structures was increased by considering two starting orientations of AFB1 for each receptor (Fig. 2).

Each of the four docking simulations generated 20 possible solutions. The conformational energy (ΔE_GM_) of the generated complexes as well as the nonbond interaction energy between the protein and the ligand were calculated. The results obtained predicted a favourable interaction between AFB1 and the active site of both VDR and RXRα (Tables 1SI and 2SI, Supporting Information). The VDR/AFB1 and RXRα/AFB1 complexes with the best compromise between conformational (ΔE_GM_) and nonbond interaction energies were chosen as the most representative ones (Table 1).

**Table 1:** Conformational and nonbond interaction energy of the selected docked complex of AFB1/VDR and AFB1/RXRα

Complex	Starting binding mode	Selected Frame	ΔE_GM_	Nonbond interaction (kcal/mol)
AFB1/VDR	I	4	0.00	−31.365
AFB1/RXRα	I	10	0.00	−32.269

The quality of the selected docked complexes was then assessed using Procheck (Laskowski et al. 1993) and it resulted comparable to that obtained for hVDR and hRXRα LBD X-ray structures (PDB IDs: 3A40 and 2P1U; Table 2).

**Table 2:** Procheck results obtained for the selected docked complexes and the human VDR and RXRα LBD X-ray structures

Structure	Residues favored regions	Residues additional allowed regions	Residues generously allowed regions	Residues disallowed regions	Ramachandran plot quality assessment
PDB ID: 3A40	93.4%	6.6%	0%	0%	Inside
PDB ID: 2P1U	95.4%	4.6%	0%	0%	Inside
AFB1/VDR	89.1%	10.0%	0.9%	0%	Inside
AFB1/RXRα	84.2%	11.9%	1.5%	2.4%	Inside

The VDR /AFB1 and RXRα/AFB1 docked complexes were then analyzed considering the experimentally determined binding modes of known agonists and antagonists of the VDR and RXRα receptors.

**Fig. 3:**
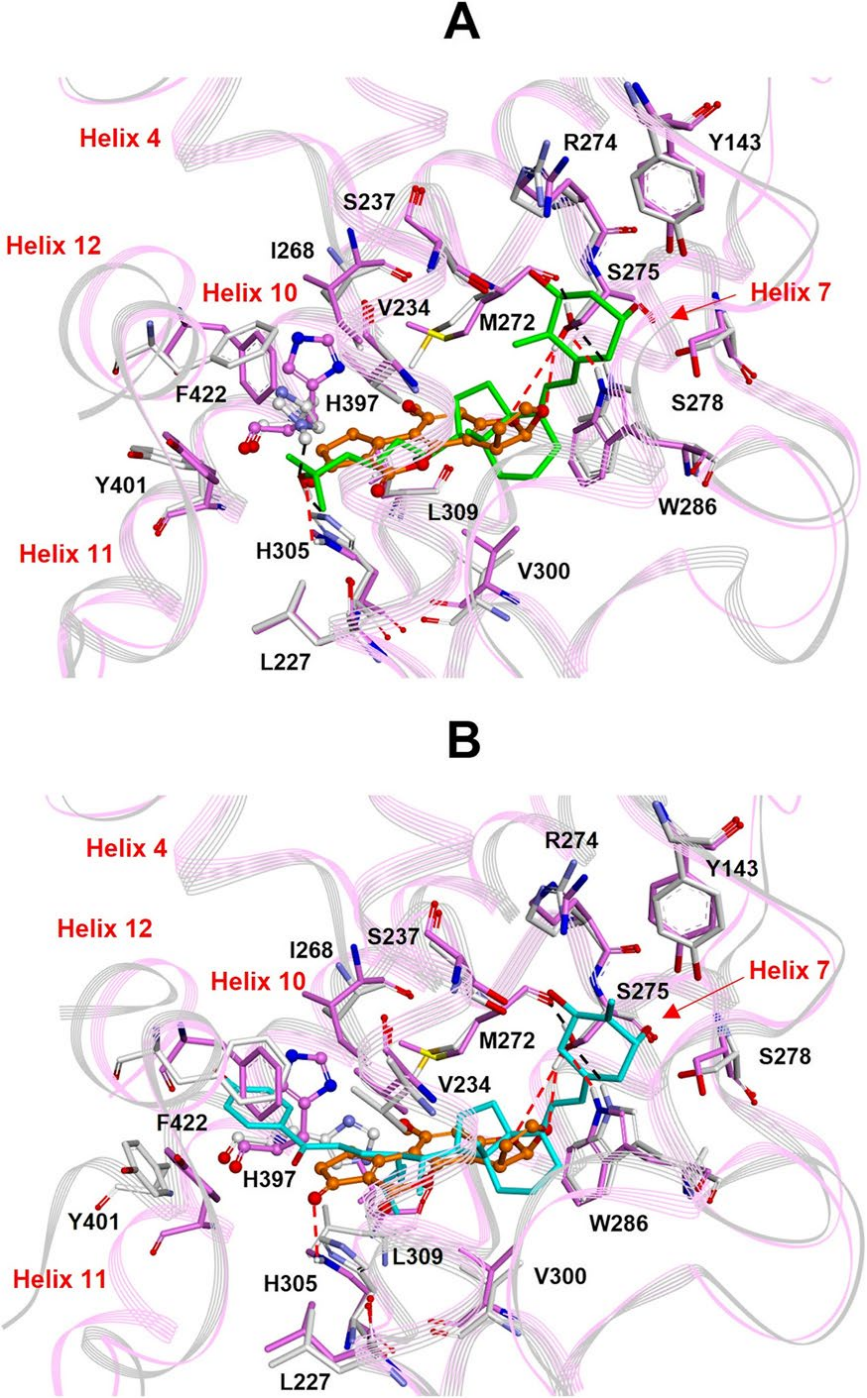
**A**: Superimposition by the Cα atoms of AFB1/VDR docked complex (AFB1: orange; VDR: pink) on the X-ray structure of 1,25(OH)_2_D_3_ in complex with VDR (1,25(OH)_2_D_3_: green; VDR: gray) (PDB ID: 1DB1). **B**: Superimposition by the Cα atoms of AFB1/VDR docked complex (AFB1: orange; VDR: pink) on the X-ray structure of the synthetic antagonist 22S-butyl-25-hydroxyphenyl-2-methylidene-19,26,27-trinor-25-oxo-1-hydroxyvitamin D3 in complex with VDR (antagonist: cyan; VDR: gray) (PDB ID: 5XPL). Heteroatoms are colored by atom type (O = red; N = blue; S = yellow). H397 is evidenced in ball & stick. Red dashed lines highlight hydrogen bonds (AFB1/VDR) or black dashed lines (agonist and antagonist/VDR). Hydrogens are omitted for clarity except those involved in hydrogen bond interactions

AFB1 binds to the VDR receptor establishing hydrophobic interactions with V234, M272, W286, and V300 similar to what was observed for the other VDR ligands (agonists and antagonists) (Fig. 3). AFB1 also establishes hydrogen bond interactions with S275 and H305 (Fig. 3A), partially reproducing the 1,25(OH)_2_D_3_ interactions. However, the formation of the hydrogen bond with S275 partially disrupts the intramolecular hydrogen bond network involving M272, S275, and W286 (Fig. 3A), which is specific to VDR among the nuclear receptor family and plays a crucial role in the correct positioning of 1,25(OH)_2_D_3_ for receptor activation (Rochel et al. 2000). In addition, contrarily to 1,25(OH)_2_D_3_ and all known agonists, AFB1 does not bind to H397 on helix 11 (Fig. 3B). This interaction strongly contributes to the dramatic stabilization of the VDR active conformation by 1,25(OH)_2_D_3_ and the other agonists (Yamamoto et al. 2000, 2006, 2007). Accordingly, AFB1 binding to VDR should not lead to receptor activation. The comparison of the selected VDR /AFB1 docking complex with the X-ray structures of the VDR/antagonist complexes supported this hypothesis. Indeed, as can be observed in Fig. 3B, the structural comparison with the synthetic competitive antagonist 22S-butyl-25-hydroxyphenyl-2-methylidene-19,26,27-trinor-25-oxo-1-hydroxyvitamin D3 (Kato et al. 2017) put in evidence that the binding of AFB1 shifts the position of H397 similarly to what observed for the VDR/antagonist X-ray complex.

**Fig. 4:**
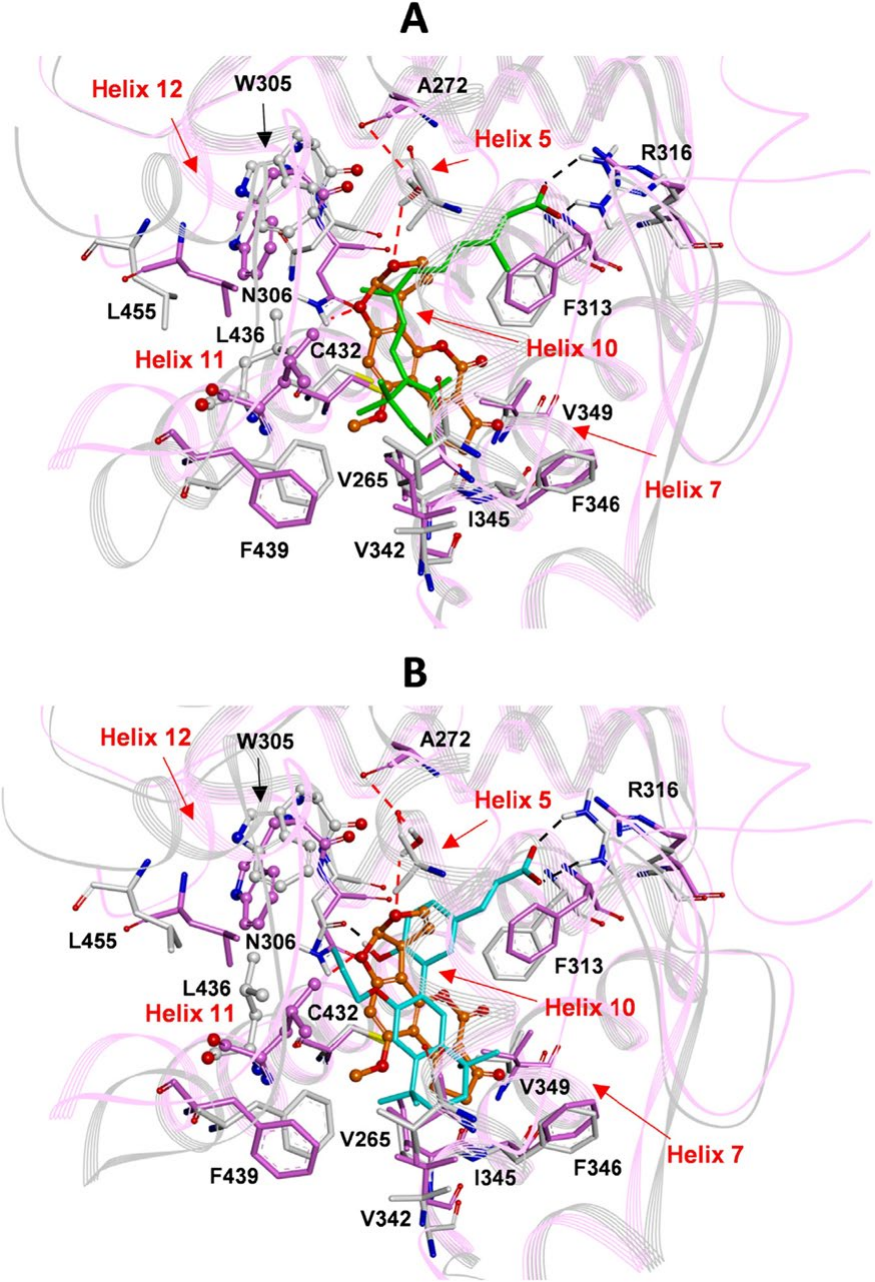
**A**: Superimposition by the Cα atoms of AFB1/RXRα docked complex (AFB1: orange; RXRα: pink) on the X-ray structure of the 9-cis retinoic acid in complex with RXRα (ligand: green; protein: gray) (PDB ID: 1FBY). **B**: Superimposition by the Cα atoms of AFB1/RXRα docked complex (AFB1: orange; RXRα: pink) on the X-ray structure of the synthetic antagonist 3-(2'-propoxy)-tetrahydronaphtyl cinnamic acid in complex with RxRα (ligand: cyan; protein: gray) (PDB ID: 2P1V). W305 and L436 are evidenced in ball & stick. Heteroatoms are colored by atom type (O = red; N = blue; S = yellow). Red dashed lines highlight hydrogen bonds (AFB1/RXRα ) or black dashed lines (agonist and antagonist/RXRα). Hydrogens are omitted for clarity except those involved in hydrogen bond interactions

Regarding the RXRα receptor, AFB1 binds to LBD, establishing hydrophobic interactions with V265, V342, I345, V349 and L436, like what was observed for the other RXRα ligands (agonists and antagonists) (Fig. 4).

Also, AFB1 establishes two hydrogen bond interactions with the RXR LBD: one with a water molecule bridged to A272 and one with N306 on helix 5 (Fig. 4A). This latter interaction caused a change in the position of W305 (Fig. 4A), a critical residue involved in the stabilization of the LBD active conformation (Iwema et al. 2007). Moreover, the binding of AFB1 shifts the position of L436 (helix 11) similar to what was observed for the antagonist 3-(2'-propoxy)-tetrahydronaphtyl cinnamic acid (Fig. 4B) (Nahoum et al. 2007). These results supported the hypothesis that AFB1, as in the case of VDR, should impair the active conformation of the LBD of RXRα.

### AFB1 negatively affects VDR expression

**Fig. 5:**
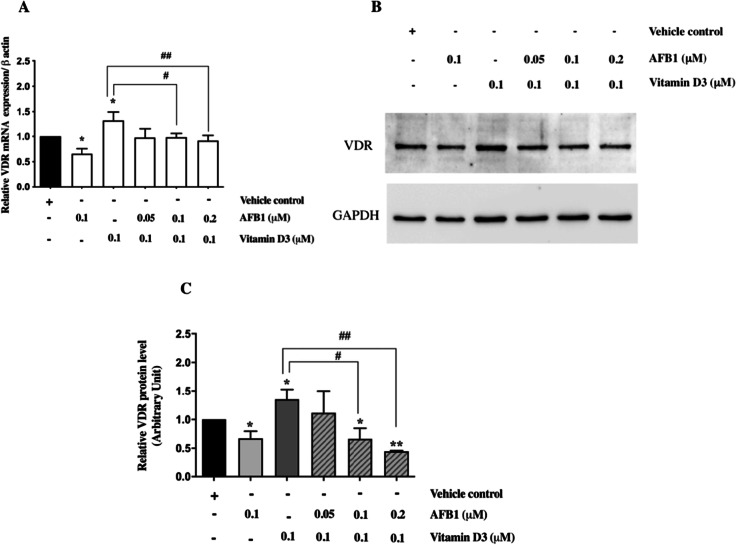
Effect of single and combined treatments with AFB1 and vitamin D3 on VDR mRNA and protein levels in Saos-2 cells. **A**: Real-time PCR analysis of VDR mRNA expression levels in Saos-2 cells treated with AFB1 (0.1 μM) or vitamin D3 (0.1 μM) for 24 h or a fixed dose of vitamin D3 (0.1 μM) and increasing amounts of AFB1 (0.05, 0.1, 0.2 μM). **B**: Western blot analysis of VDR expression levels in Saos-2 cells treated with AFB1 and vitamin D3 as described above. The figure shows representative results of three independent experiments. **C**: Densitometric analysis of western blot results performed by Image J software. Differences were considered significant when *p* < 0.05 and highly significant when *p* < 0.0001. *p < 0.05, **p < 0.0001 versus vehicle control (calculated as fold change relative to vehicle cells, arbitrarily set as 1); #*p* < 0.05, ##*p* < 0.0001 treatment with vitamin D3_3_ (0.1 μM) versus combined treatment with increasing doses of AFB1

Based on these computational data, we next performed biochemical and molecular studies to investigate the putative antagonist role of AFB1 on the vitamin D mechanism of action. Previously, we had demonstrated that AFB1 treatment in osteosarcoma cell line Saos-2 is associated with down-modulation of VDR expression at RNA and protein levels (Costanzo et al. 2015). Therefore, to explore the hypothesis that AFB1 antagonizes the action of vitamin D, the effects of single and combined treatments with AFB1 and vitamin D3 were evaluated on VDR mRNA and protein levels. As expected, vitamin D3 treatment determined a significant increase in VDR expression levels. Conversely, in agreement with our previous report (Costanzo et al. 2015), AFB1 exposure strongly reduced VDR expression both at RNA and protein levels. Co-treatments with vitamin D3 and increasing amounts of AFB1 revealed that AFB1 exposure progressively reduces VDR expression in a dose-dependent manner. These results indicate that AFB1 can counteract the positive effect of vitamin D on VDR expression and are consistent with an antagonistic role of AFB1 against vitamin D3 activity (Fig. 5).

### AFB1 impairs transcription complexes assembly on VDR gene regulatory elements

**Fig. 6:**
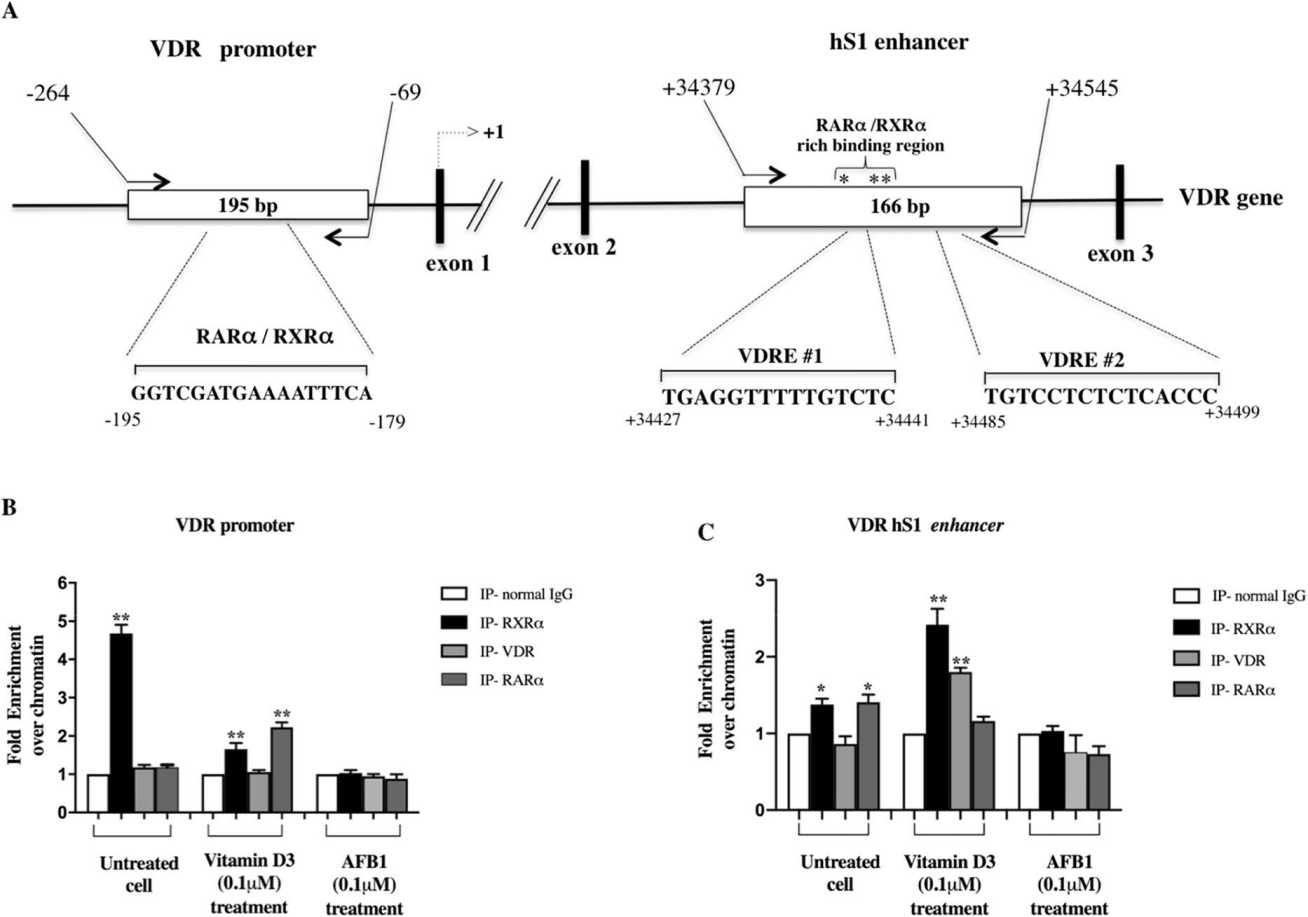
ChIP analysis on VDR promoter and hS1 intronic enhancer in Saos-2 cells treated with AFB1 or vitamin D3. **A**: Schematic representation of the VDR gene structure. The exonic regions are depicted as black boxes, white boxes indicate the VDR and RXRα/RARα responsive elements. Arrows mark the positions of the oligonucleotides used for the quantitative Real time PCR (qPCR). ChIP assays were performed with anti-RXRα, anti-VDR or anti-RARα antibodies in Saos-2 cells treated with AFB1 or vitamin D3 (0.1 μM) for 6 h. Immunoprecipitation with non-specific IgG was used as negative control. Non-immunoprecipitated chromatin was used as total input control. The immunoprecipitated chromatin was analyzed by qPCR to evaluate RXRα, VDR and RARα binding to VDR promoter and hS1 enhancer regions as depicted in panel B and panel C, respectively. Results are representative of two independent experiments. Differences were considered significant when *p* < 0.05 and highly significant when *p* < 0.0001. *p < 0.05, **p < 0.0001 versus each negative control, calculated as fold change relative to IgG and arbitrarily set as 1

We next asked whether the molecular mechanisms underlying VDR down-modulation mediated by AFB1 exposure could be related to impaired assembly of vitamin D3-dependent transcriptional complexes that include the involvement of VDR and retinoid receptors RXRα and RARα (Pike et al. 2014; Brtko and Dvorak 2020). As a starting point, in order to evaluate the effects of AFB1 on VDR and/or retinoid receptors recruitment at vitamin D responsive elements (VDRE), we performed Chromatin Immunoprecipitation (ChIP) analysis on a VDR intragenic enhancer region (hS1) containing VDREs, 5’-flanked by retinoid receptors binding sites actively involved in VDR transcriptional autoregulation (Fig. 6A) (Zella et al. 2006). To this aim, Saos-2 cells were treated with 0.1 μM AFB1 or 0.1 μM vitamin D3 and 6 h after treatment, the chromatin was immunoprecipitated with RXRα, RARα, and VDR antibodies and analyzed by quantitative real-time PCR. We observed significant signals corresponding to both RXRα and RARα occupancy on this region in untreated cells (Fig. 6C), suggesting the recruitment of these receptors in the absence of vitamin D signaling. On the other hand, as expected, vitamin D treatment was accompanied by increased enrichment in RXRα and VDR with a concomitant reduction in RARα occupancy, consistent with the recruitment of the RXRα/VDR heterodimer in this regulatory region. Interestingly, following AFB1 treatment, we observed no enrichment of either RXRα, VDR, or RARα with respect to the IgG negative control (blank control), thus demonstrating that AFB1 caused disruption of all these DNA–protein interactions in this genomic region (Fig. 6C). The result of ChIP assays indicates that AFB1 can interfere with the transcriptional machinery promoted by vitamin D3 signaling (Gocek et al. 2007). Notably, these findings agree with our docking data indicating that AFB1 binding to VDR LBD could impair the activation of this receptor.

Since docking data had shown that AFB1 could bind RXRα in the LBD region and impair receptor activation, we asked whether the recruitment of other transcriptional complexes requiring RXRα for transcriptional activation might be similarly affected. To address this question, we firstly performed an in silico analysis using JASPAR (Fornes et al. 2020) (profile score threshold = 75%) to search for the presence of putative responsive elements to retinoid receptors in the -960 VDR promoter region that, according to literature data (Zella et al. 2006), does not contain VDRE consensus sequences. More in detail, we focused our analysis on the fragment from -264 to -69 bp and found putative RXR-based heterodimers responsive elements, including RXRα-RARα (Fig. 6 and Fig. 1SI). ChIP assays were performed to evaluate the effect of vitamin D3 and AFB1 on the recruitment of RXRα and RARα in this regulatory region. Results showed a significant signal corresponding to RXRα occupancy on this region in untreated cells (Fig. 6B), suggesting its recruitment on this VDR promoter region in the absence of vitamin D stimulus.

Conversely, vitamin D3 treatment was accompanied by enrichment in RARα occupancy and decreased RXRα binding, consistent with the recruitment of RXRα/VDR heterodimers following vitamin D exposure. As expected, no VDR enrichment was found following vitamin D3 treatment in this region (Fig. 6B). Noteworthy, exposure to AFB1 resulted in both RXRα and RARα binding displacement, indicating that AFB1 can impair the recruitment of RXRα/RARα complexes elicited by vitamin D treatment. Importantly, these results provide experimental evidence of the possible antagonistic role of AFB1 on RXRα activation, as illustrated by docking studies.

**Fig. 7:**
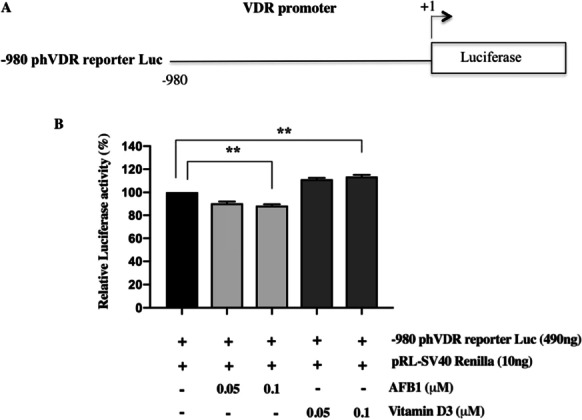
Luciferase reporter assay of VDR promoter activity in Saos-2 cells treated with AFB1 and vitamin D3. **A**: Schematic representation of the reporter plasmid (-960 ph VDR/Luc) containing a 960 bp fragment of human proximal VDR promoter cloned upstream of the luciferase reporter gene. **B**: Reporter luciferase activity was evaluated in cells transfected with the -960 ph VDR/Luc plasmid and treatment with two different doses of AFB1 and vitamin D3 (0.05–0.1 μM) for 24 h. Data were normalized to Renilla luciferase activity (internal control) and values were expressed in percentages as the mean ± standard deviation (SD) of three independent experiments. Differences were considered significant when *p* < 0.05 and highly significant when *p* < 0.0001. *p < 0.05, **p < 0.0001 versus mock control (calculated as fold change relative to mock cells, arbitrarily set at 100%)

Finally, in light of these results, we performed gene reporter assays on a plasmid vector containing the human proximal VDR promoter region cloned upstream of the luciferase reporter gene (pVDR/Luc) to assess the contribution of this region to vitamin D-dependent up-regulation of VDR expression and to demonstrate that AFB1 binding impairs RXRα activation and, consequently, its transcriptional activity. To this aim, Saos-2 cells transfected with pVDR/Luc vector were treated with two different doses of AFB1 and vitamin D3 (0.05 μM and 0.1 μM) for 6 h, and then the luciferase assays were performed. Whereas vitamin D3 increased the VDR promoter activity, AFB1 treatment significantly decreased the luciferase activity, thus further reinforcing our previous data indicating AFB1 as a down-modulator of VDR expression. Notably, our data also support the hypothesis raised from docking data that the binding to RXRα LBD could block its activation and contribute to down-regulate VDR expression (Fig. 7).

Taken as a whole, our results indicate that AFB1 can interfere with vitamin D-mediated transcriptional activation of VDR expression by impairing the formation and recruitment of both RXRα/RARα and RXRα/VDR protein complexes, thus providing experimental evidence to the docking data predicting molecular interactions between AFB1 and VDR or RXRα (Figs. 3 and 4).

### AFB1 affects subcellular distribution of VDR and RXR*α*

**Fig. 8:**
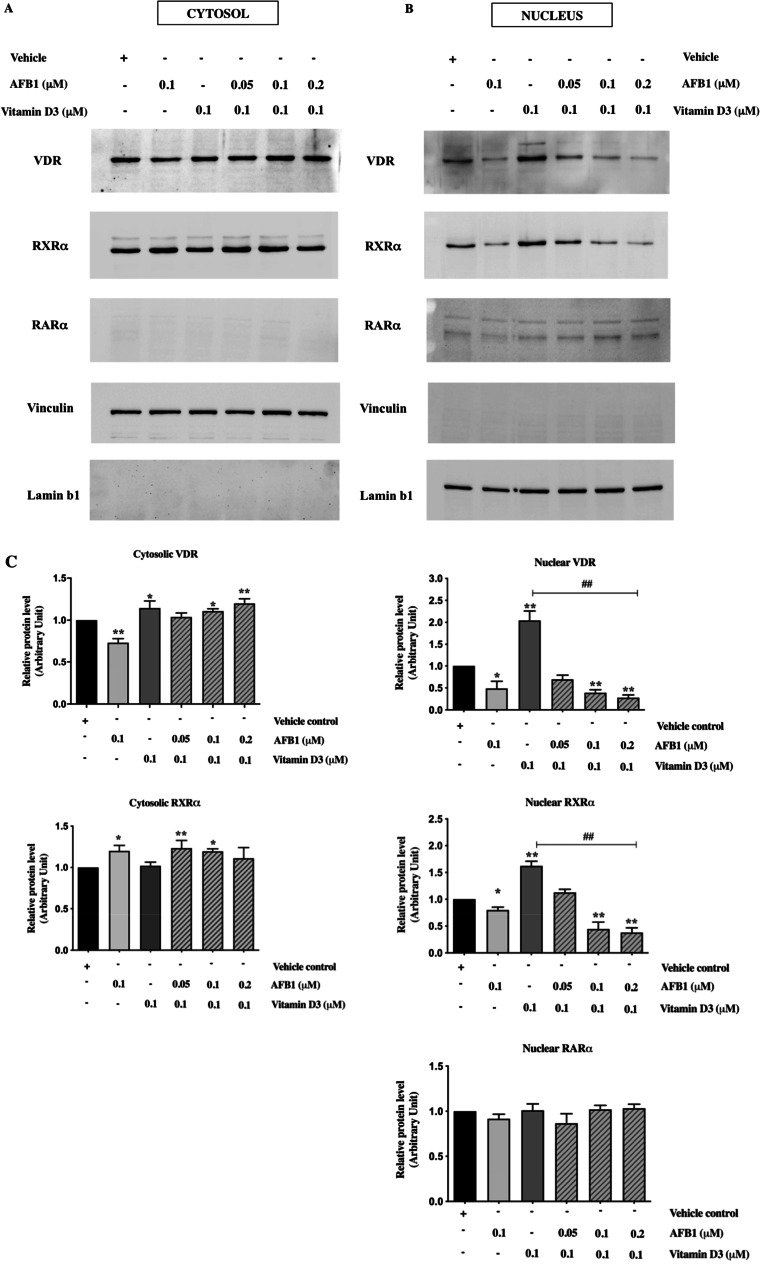
Evaluation of VDR, RXRα, and RARα subcellular distribution in AFB1 and vitamin D3 treated Saos-2 cells. **A**: Western blot analysis of RXRα, VDR and RARα expression levels in cytosolic extracts obtained after a single exposure to AFB1 (0.1 μM) and vitamin D3 (0.1 μM) for 24 h or combined treatments with vitamin D3 (0.1 μM) and increasing AFB1 amounts (0.05, 0.1 and 0.2 μM). **B**: Western blot analysis of nuclear extracts performed with anti-RXRα, anti-VDR, and anti-RARα antibodies following single and combined treatment with AFB1 and vitamin D3 as described above. The purity of cytosolic and nuclear extracts was checked by anti-vinculin and lamin B1 antibodies. The figure shows representative results quantified from three independent experiments. **C**: Densitometric analysis of western blot results was performed for each immunoblot using Image J software, and bands were normalized to vinculin used as a loading control for the cytosol fraction and Lamin B1 as a loading control for the nuclear fraction. Differences were considered significant when *p* < 0.05 and highly significant when *p* < 0.0001. *p < 0.05, **p < 0.0001 versus mock control (calculated as fold change relative to mock cells, arbitrarily set as 1); #*p* < 0.05, ##*p* < 0.0001 single treatment with vitamin D3 (0.1 μM) versus combined treatment with increasing doses of AFB1

According to the evidence that the mechanism of action of vitamin D includes regulation of nucleo-cytoplasmic shuttling of VDR and RXR, with nuclear import of RXR-VDR heterodimers being mediated preferentially by VDR and controlled by the VDR ligand (Yasmin et al. 2005), we chose to investigate more in detail the molecular mechanisms underlying AFB1 interference against vitamin D signaling by examining the subcellular localization of VDR, RXRα, and RARα in Saos-2 cells treated with vitamin D3 and/or AFB1. Western blot analysis on cytosol (Fig. 8A) and nuclear (Fig. 8B) protein extracts showed, as expected, a slight but significant increase of VDR cytosolic levels (Fig. 8A, lane 3) along with a more dramatic increase of its nuclear fraction following vitamin D3 treatment (Fig. 8B, lane 3), expectedly due to both vitamin D-dependent transcriptional activation and nuclear translocation of VDR (Yasmin et al. 2005; Fadel et al. 2020). Following vitamin D3 treatment, RXRα cytosolic levels did not significantly change (Fig. 8A, lane 3). In contrast, they were consistently increased in the nucleus (Fig. 8B, lane 3) in agreement with the notion that VDR mediates the nuclear translocation of the VDR-RXRα heterodimer elicited by vitamin D signaling (Fornes et al. 2020; Yasmin et al. 2005). In this context, it is interesting to note that, although RXRα and VDR translocate into the nucleus by distinct pathways, vitamin D triggers the recruitment of RXR-VDR heterodimers to the VDR nuclear import carrier (importin α) to promote their nuclear translocation (Fornes et al. 2020; Yasmin et al. 2005).

On the contrary, AFB1 treatment was accompanied by reduced VDR levels at cytosolic and nuclear compartments, consistent with the evidence that AFB1 impairs both VDR transcription activation and nuclear translocation. In the case of RXRα, our results showed increased levels of the cytosolic fraction and decreased nuclear levels indicating that AFB1 exposure elicits cytosolic retention of RXRα, thus further supporting docking data showing mechanisms of inhibitory activity of AFB1 on RXRα activation. Interestingly, combined treatments with 0.1 μM vitamin D and increasing doses of AFB1 ranging from 0.05 to 0.2 μM showed that AFB1 could impair the effects triggered by vitamin D on both VDR and RXRα subcellular distribution in a dose-dependent manner. As shown in Fig. 8, analysis performed on nuclear and cytosolic extracts indicated that AFB1 can retain both VDR and RXRα in the cytosolic fraction and counteract in a dose-dependent manner their nuclear translocation elicited by vitamin D3.

As regards RARα, its localization is predominantly nuclear even in the absence of its ligand (Xu et al, 2017). Consistent with these observations, our western blot analysis detected appreciable hybridization signals for RARα only in the nuclear fraction, whose levels are modified neither by vitamin D3 nor by AFB1 treatment (Fig. 8).

**Fig. 9:**
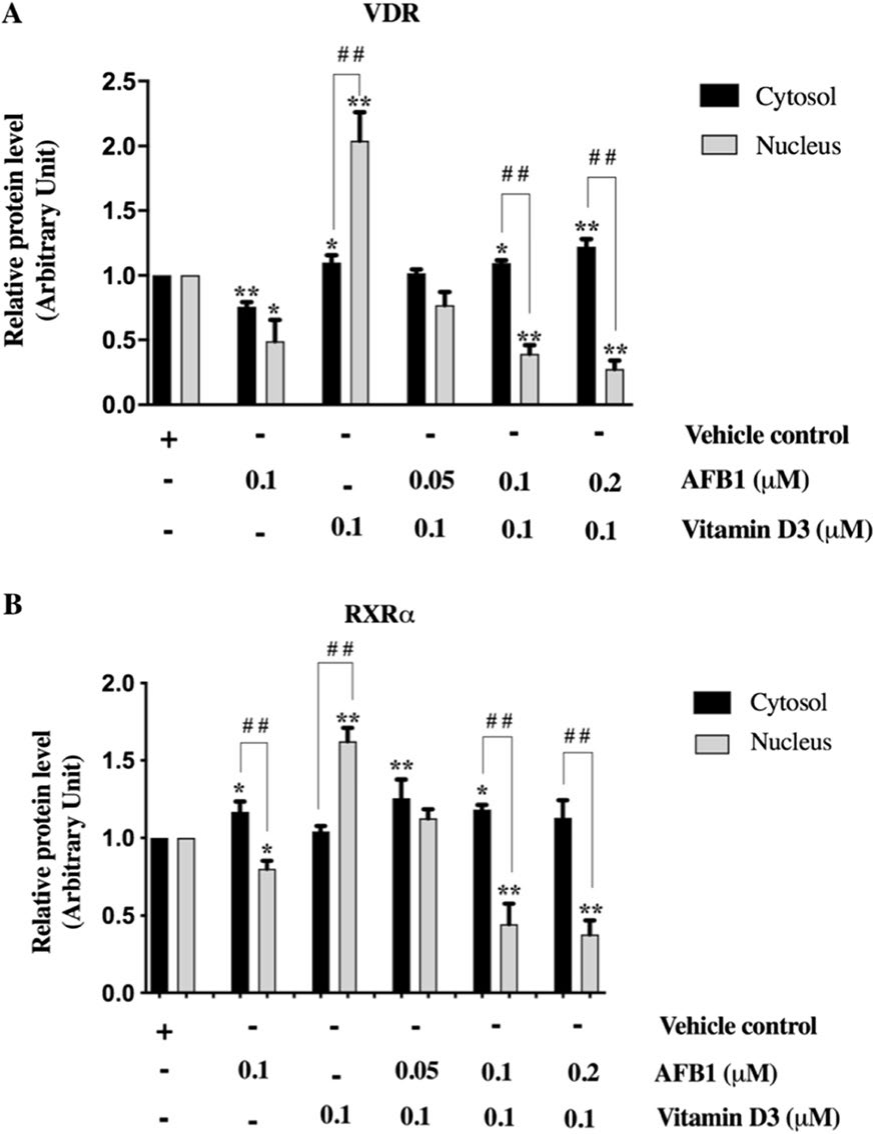
Evaluation of VDR and RXRα subcellular distribution in AFB1 and vitamin D3 treated Saos-2 cells. **A**: Comparison of VDR cytosolic and nuclear expression levels in Saos-2 cells after single or combined treatment with AFB1 and vitamin D3. **B**: Comparison of RXR-α cytosolic and nuclear expression levels in Saos-2 cells after single or combined treatment with AFB1 and 1 vitamin D3. The graphs show representative results of three independent experiments analyzed by Image J software. Differences were considered significant when *p* < 0.05 and highly significant when *p* < 0.0001. *p < 0.05, **p < 0.0001 versus mock control calculated as fold change relative to mock cells, arbitrarily set as 1

To better illustrate variations in subcellular distribution of both VDR and RXRα, we compared their cytosol and nuclear levels (Fig. 9). As expected, we found that vitamin D affects the relative cytosol/nuclear subcellular distribution of both VDR and RXRα by increasing their nuclear fractions. Conversely, AFB1 exerts an opposite effect on both receptors. Furthermore, co-treatments with vitamin D and increasing doses of AFB1 dramatically reduce the nuclear fraction of both receptors in agreement with its antagonistic role against vitamin D, as highlighted by computational analysis. As expected in these conditions, we observed an increase in the cytosolic fraction of VDR, whereas the RXRα cytosolic fractions were not affected in a similar manner, probably due to proteasome-dependent degradation of retinoid signaling (Fig. 9) (Rodriguez et al. 2019).

These results are consistent with different inhibitory mechanisms played by AFB1 on vitamin D3 signaling, including VDR transcriptional regulation and nuclear translocation of VDR and RXRα. Importantly, these data are in complete agreement and provide experimental evidence to the docking data indicating an antagonist effect of AFB1 on both VDR and RXRα activation.

## Discussion

**Fig. 10:**
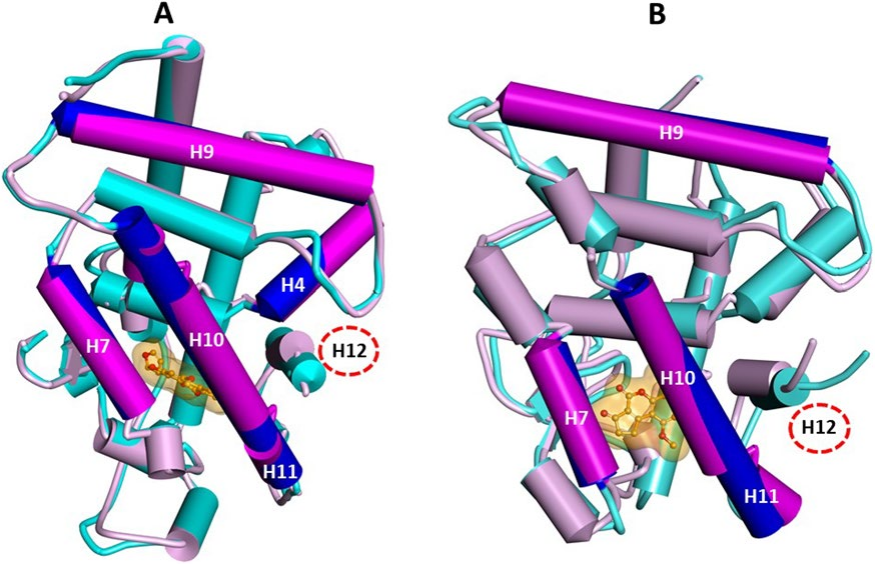
**A**: Superimposition by the Cα atoms of AFB1/VDR docked complex (AFB1: orange; VDR: pink) on the starting hVDR LBD conformation (PDB ID: 3A40; VDR: cyan). **B**: Superimposition by the Cα atoms of AFB1/RXRα docked complex (AFB1: orange; RXRα: pink) on the starting hRXRα LBD conformation (PDB ID: 2P1U; protein: cyan). Helices are displayed as wide cylinders, beta-sheets as arrows, and coil and turn regions as tubes. H12 is evidenced with a red dashed circle. The helices involved in the heterodimerization are colored in blue in the starting structures and in magenta in the AFB1/VDR and AFB1/RXRα complexes. The ligands are displayed in ball & stick, and their solvent-accessible surfaces are shown. Heteroatoms are colored by atom type (O = red)

The transactivation of VDR is characterized by a series of sequential molecular events, such as ligand binding, dimerization with the partner receptor, translocation to the nucleus, recruitment of co-regulators, and binding to DNA. Structural and biochemical results indicated that transactivation occurs through a local conformational change of helices 10, 11, and 12 in the LBD (Kakuda et al. 2010; Kato et al. 2016). Indeed, upon agonist binding, the loop between helices 10 and 11 converts to form sequential helix 10/11 and helix 12 folds back, adopting the proper conformation for the interaction with the partner protein(s) (Fig. 2SI) (Anami et al. 2016). The dimerization interface between VDR and RXRα involves helices 4, 7, 9, 10, and 11 of VDR and helices 7, 9, 10, and 11 of RXRα (Asano et al. 2016; Orlov et al. 2012; Zhang et al. 2011) . Mutagenesis studies demonstrated that the interaction with the histidine (H397) residue placed at the C-terminal of the VDR LBD, and making part of the active site, plays a crucial role in the activation mechanism (Yamamoto et al. 2000, 2006, 2007). Indeed, the hydrogen bond with H397 is a crucial interaction established by all known agonists essential for ligand binding and transactivation (Rochel et al. 2000; Yamamoto et al. 2000, 2006). Some VDR antagonists still interact with H397 but induce changes in the conformation of the helix 6/loop 6 − 7/helix 7 regions inhibiting heterodimerization with RXRα (Kato et al. 2016). A second category of VDR antagonists do not interact with H397 and shifts its position (Fig. 3B, PDB ID: 5XPL *vs.* Figure 3A, PDB ID: 1DB1), destabilizing the correct folding of helices 10/11 and 12, necessary for receptor heterodimerization and activation (Kato et al. 2017). According to our docking studies, AFB1 is hydrogen-bonded to H305 stabilizing the helix 6/loop 6 − 7/helix 7 region, while it cannot establish any interaction with H397 (Fig. 3). These results suggest that AFB1 may act as a VDR antagonist belonging to the second category. Moreover, it is noteworthy that the starting agonist-bound receptor conformation (PDB ID: 3A40), which is very similar (Cα RMSD: 0.259 Å) to the structure of hVDR LBD in complex with the endogenous ligand 1,25(OH)_2_D_3_ (PDB ID: 1DB1; Fig. 3SI A), was significantly perturbed (Fig. 10A). In particular, AFB1 binding to the VDR LBD disrupts the sequential helix 10/11, prevents the proper helix 12 folding for receptor activation, and moves helices 7 and 9, thus affecting the molecular surface involved in the dimerization with RXRα.

On the other hand, RXR presents different structural dynamics of the AF-2 domain, which can correctly fold for hetero-dimerization with VDR in the absence of 9-*cis*-RA (Evans and Mangelsdorf 2014). Expressly, previous studies indicated that 9-cis-RA inhibits, whereas D3 strengthens, the interactions between the receptors and that the heterodimer is maximally stabilized in the presence of both ligands (Dong and Noy 1998; Thompsonet al. 1998). Mutagenesis studies demonstrated that the mutation of W305, a residue conserved in all RXR receptors and placed on helix 5 of their LBDs, affords a dramatic loss-of-function equivalent to helix 12 deletion. Hence, this residue plays a vital role in stabilizing the active conformation of RXRα LBD (Iwema et al. 2007; Kojetin et al. 2015). Moreover, crystal structures revealed that RXRα antagonists impair helix12 mobility by modifying the conformation of L436 on helix 11, which is involved in a stabilizing interaction with helix 12 (Nahoum et al. 2007). According to our docking studies, as reported in Fig. 10B, where the AFB1/RXRα LBD docked complex is superimposed on the starting protein conformation (PDB ID: 2P1U), AFB1 shifts the position of W305 and L436, disrupting the sequential helix 10/11 by binding to RXRα active site, thus preventing helix 12 folding necessary for receptor activation and shifting helices 7, 9, and 10 involved in the heterodimerization with VDR. It has to be underlined that, also in this case, the hRXRα LBD complex with the agonist 3-(2'-ethoxy)-tetrahydronaphtyl cinnamic acid (PDB ID: 2P1U), used as starting structure in docking calculations, is very similar to the structure of hRXRα LBD in complex with the endogenous ligand 9-*cis*-RA (PDB ID: 1FBY; Cα RMSD: 0.895 Å; Fig. 3SI B).

The results of our docking simulation suggest that AFB1 can bind to both VDR and RXRα LDBs, establishing molecular interactions and inducing conformational changes similar to those shown by competitive antagonists. To provide experimental evidence to these observations, expression studies were performed in the osteosarcoma cell line Saos-2 showing that AFB1 can counteract the positive effect of vitamin D3 on VDR expression, therefore supporting the antagonistic role of AFB1 against Vitamin D activity (Fig. 5). Furthermore, by chromatin immunoprecipitation experiments, we demonstrated that AFB1 prevents the formation of protein complexes containing VDR and/or RXRα receptors at different regulation loci on the VDR gene, thus indicating that AFB1 impairs transactivation activity mediated by vitamin D3 (Fig. 6). Finally, our experimental results evidenced that AFB1 affects vitamin D-induced transcriptional regulation and the nuclear translocation mechanisms induced by vitamin D.

**Fig. 11:**
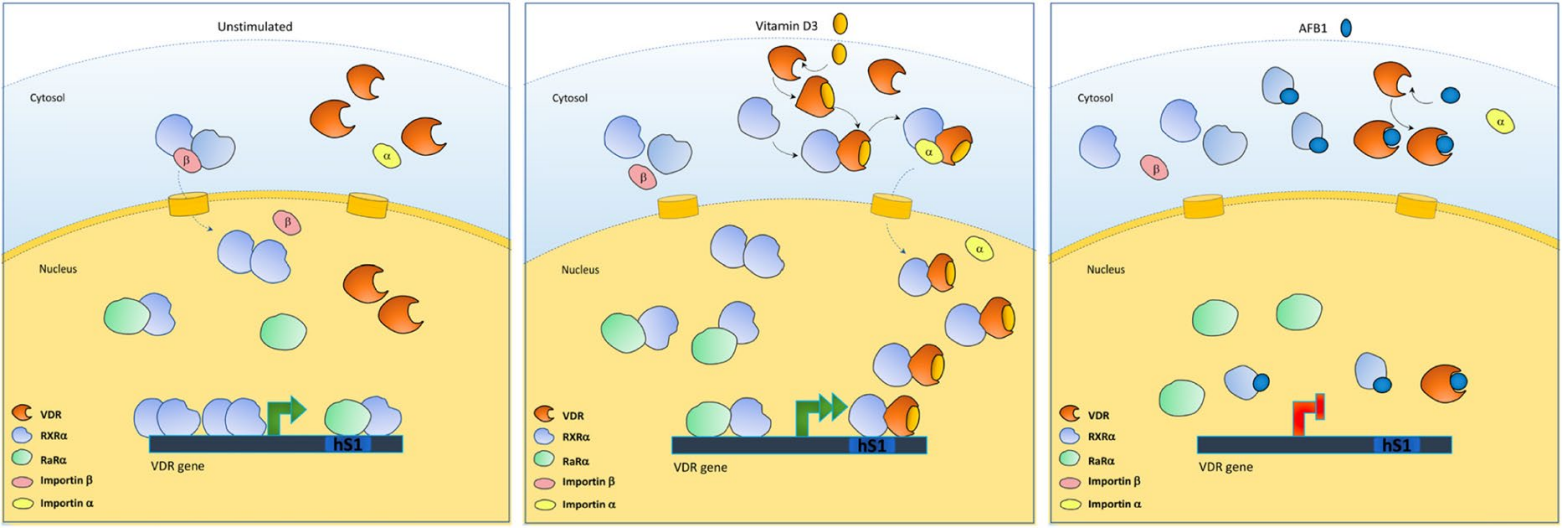
Schematic representation of the mechanism of action of AFB1 in impairing vitamin D3 activity

RXRα and VDR translocate into the nucleus by distinct pathways (Yasmin et al. 2005). The nuclear import of RXRα and VDR is mediated by importin β and importin α, respectively. In particular, VDR recruits RXR-VDR heterodimers to importin α and mediates nuclear import of the heterodimers in response to vitamin D. On the other hand, importin β binding and nuclear import of RXRα, as homodimers, are modestly enhanced by 9-cis-RA. Our results clearly show that AFB1 can affect the nuclear translocation mechanisms induced by vitamin D3, thus favouring cytosolic retention of VDR and RXRα (Figs. 8 and 9). These results provide further experimental evidence to docking data highlighting the inhibitory effect of AFB1 on VDR and RXRα activation through its antagonistic binding to the LBD domains of both receptors. Conversely, it is to be noted that RARα has predominantly a nuclear localization even in the absence of its ligand (Xu et al. 2017). Therefore, as expected in this case, AFB1 affects neither its subcellular localization nor mechanisms of cytoplasmic-nuclear shuttling and it remains confined to the nucleus. As a whole, our study indicates that AFB1 affects vitamin D signaling by at least two mechanisms, including regulation of transactivation activity and cellular content of VDR and RXRα receptors (Fig. 11).

The amount of RXR is limited within the cells, so there is a dynamic competition among RXRα heterodimerization partners (in the absence of agonists RARα > VDR), and the binding of their specific agonist increases the affinity of a given receptor favoring its heterodimerization with RXRα (Fadel et al. 2020). Intriguingly, our findings also raised the hypothesis that AFB1 may also affect 9-cis-RA signaling through inhibition of RXRα activation and RXRα-RARα heterodimerization and allowed us to shed light on yet unexplored mechanisms of toxicity mediated by AFB1 on vitamin D and retinoid receptors. Our study thus provides the first mechanistic evidence of AFB1 as immune response and endocrine disruptor and is instrumental to define a link connecting the onset of adverse health outcomes such as growth retardation, malnutrition, immunosuppression, infertility, and carcinogenicity to AFB1 exposure.

Therefore, in light of these findings, given the broad range of target genes that mediate the pleiotropic effects of RA and vitamin D on cell growth, differentiation and apoptosis, future perspectives should include investigations into genome-wide transcriptional profiling for a deeper understanding of the basis of AFB1 toxicity and to stimulate more effective preventive and control actions by food safety authorities.

## Material and Methods

### Molecular modeling

Molecular modeling calculations were performed on E4 Server Twin 2 × Dual Xeon-5520, equipped with two nodes. Each node: 2 × Intel® Xeon® QuadCore E5520-2.26Ghz, 36 GB RAM. The molecular modeling graphics were carried out on a personal computer equipped with Intel(R) Core (TM) i7-4790 processor and SGI Octane 2XR12000 workstations.

### Analysis of structural properties of Aflatoxin B1

The experimentally determined structures of Aflatoxin B1 (CSD codes: AFLATC and AFLATM) were downloaded from the Cambridge Structural Database (CSD) using the CSDS (Cambridge Structural Database System) software Conquest 1.18. The apparent pKa values of aflatoxin were calculated using ACD/Percepta software. (ACD/Percepta software, version 2017.1.3, Advanced Chemistry Development, Inc., Toronto, ON, Canada, 2017; http://www.acdlabs.com.) The compound was considered neutral in all calculations performed because of the percentage of neutral/ionized forms computed at pH 7.4 (physiological value) using the Handerson–Hasselbalch equation. The compounds were assigned atomic potentials and partial charges using the CVFF force field (Dauber-Osguthorpe et al. 1998).

### Structural and bioinformatic analysis

The experimentally determined structures of the LBD of VDR (PDB IDs: 1DB1, 1IE8, 1IE9, 1KB2, 1KB4, 1KB6, 1S0Z, 1S19, 1TXI,1YNW, 2HAM, 2HAR, 2HAS, 2HB7, 2HB8, 3A2I, 3A2J, 3A3Z, 3A40, 3A78, 3AUQ, 3AUR, 3AX8, 3AZ1, 3AZ2, 3AZ3, 3B0T, 3CS4, 3CS6, 3KPZ, 3M7R, 3OGT, 3P8X, 3TKC, 3VHW, 3W0A, 3W0C, 3W0Y, 3WGP, 4G2I, 4ITE, 4ITF, 1RJK, 1RK3, 1RKG, 1RKH, 2O4J, 2O4R, 2ZFX, 2ZL9, 2ZLA, 2ZLC, 2ZMH, 2ZMI, 2ZMJ, 2ZXM, 2ZXN, 3A2H, 3AFR, 3AUN, 3VJS, 3VJT, 3VRT, 3VRU, 3VRV, 3VRW, 3VT3, 3VT4, 3VT5, 3VT6, 3VT7, 3VT8, 3VT9, 3VTB, 3VTC, 3VTD, 3W0G, 3W0H, 3W0I, 3W0J, 3W5P, 3W5Q, 3W5R, 3W5T, 3WT5, 3WT6, 3WT7, 5XPL) and RXRα (PDB IDs: 1BY4, 1FBY, 1FM6, 1FM9, 1G1U, 1G5Y, 1K74, 1LBD, 1MV9, 1MVC, 1MZN, 1R0N, 1RDT, 1XDK, 1XLS, 1XV9, 1XVP, 2ACL, 2P1T, 2P1U, 2P1V, 2ZXZ, 2ZY0, 3DZU, 3DZY, 3E00, 3E94, 3FAL, 3FC6, 3FUG, 3H0A, 3KWY, 3NSP, 3NSQ, 3OAP, 3OZJ, 3PCU, 3R29, 3R2A, 3R5M, 3UVV, 4J5W, 4K4J, 4K6I, 4M8E, 4M8H, 4N5G, 4N8R, 4NQA, 4OC7, 4POH, 4POJ, 4PP3, 4PP5, 3A9E), were downloaded from the Protein Data Bank (PDB; http://www.rcsb.org/pdb/).

All the structures were superimposed by sequence alignment and ligand-induced protein conformational changes as well as ligand–protein interactions were analyzed (Biopolymer and Homology module of Insight 2005; Accelrys, San Diego). In particular, hydrogen atoms were added (pH of 7.2) and the interactions with all protein amino acids and water molecules having at least one atom within a 5 Å radius from any given ligand atom were monitored.

The best solved (i.e., more complete and highest resolution) structure of the LBD of hVDR (in complex with the agonist 2alpha-methyl-AMCR277B; resolution 1.45 Ǻ; PDB ID: 3A40) (Antony et al. 2010) was selected as starting protein conformation in docking studies while two very similar (Cα RMSD = 1.14 Ǻ) and high resolution (≤ 2.20 Ǻ) structures of hRXRα LBD were selected to model the full-length hRXRα LBD (see below).

The presence of RXR-based heterodimer responsive elements on the VDR promoter were predicted using the database of transcription factor binding profiles JASPAR (http://jaspar.genereg.net). In particular, the fragment from -264 to -69 of the VDR promoter was analyzed using the following position frequency matrices (PFMs): MA0074.1 (RXRα-VDR), MA0065.1 (PPARγ- RXRα), MA0115.1 (NR1H2-RXRα) MA0159.1 (RARα-RXRα), MA1146.1 (NR1H4-RXRα), MA1147.1 (NR4A2-RXRα), MA1148.1 (PPARα-RXRα) and MA1149.1 (RARα-RXRG). A relative profile score threshold of 75 was used for the selection.

### Modeling of hRXRα ligand-binding domain (LBD)

As above reported, the following template structures were selected to build the molecular model of full length RXRα LBD. The X-ray structure of hRXRα LBD in complex with the agonist 3-(2'-ethoxy)-tetrahydronaphtyl cinnamic acid (Nahoum et al. 2007) (PDB ID 2P1U; resolution: 2.20 Ǻ), lacking residues 243–263, was selected as main template structure while the missing loop was modeled using the X-ray structure of the heterodimer RXRα/PPARγ (Gampe et al. 2000) (PDB ID: 1FM6; resolution 2.10 Ǻ). Ligand molecules were removed and the sequence of 2P1U and 1FM6 were aligned with hRXRα sequence downloaded from the UniProtKB/Swiss-Prot Data Bank (http://www.uniprot.org; entry P19793) by using the Multiple-Alignment algorithm (Homology module, Insight 2005, Accelrys, San Diego). Structurally conserved regions (SCR) were defined as: i) residues 229 − 242 and 264–458 of 2P1U and ii) residues 243–263 of 1FM6. The coordinates of the SCR were transferred to the hRXRα sequence by the SCR-Assign Coords procedure (Homology; Accelrys, San Diego).

The obtained homology model of RXRα LBD was completed inserting the water molecules of RXRα experimentally determined structure (PDB ID: 2P1U) through the UnMerge and Merge commands (Biopolymer module, Insight 2005, Accelrys, San Diego). Atomic potentials and partial charges were assigned using the CVFF force field. The homology model was then subjected to a total energy minimization within Insight 2005 Discover-3 module (Steepest Descent algorithm, maximum RMS derivative = 1 kcal/Å; ε = 1; Cell Multipole method for non-bond interactions (Ding et al. 1992). Only the region aa239 − 271 was left free to move during the minimization, whereas the structurally conserved regions (SCRs) of RXRα LBD were fixed to avoid unrealistic results. The final model was checked by using the Struct_Check command of the ProStat pulldown in the Homology module to verify the correctness of the geometry optimization procedure before moving to the next step. Checks included φ, ψ, χ1, χ2, χ3, and ω dihedral angles, Cα virtual torsions, and Kabsch and Sander main chain H-bond energy evaluation. The RXRα LBD homology model was used for successive dynamic docking studies.

### Docking studies on human VDR and RXRα receptors in complex with Aflatoxin B1

The homology model of the full-length hRXRα LBD and the best solved structure of hVDR LBD (PDB ID: 3A40) were employed as starting protein structures in dynamic docking studies. The ligand of 3A40 was removed and atomic potentials and partial charges were assigned using the CVFF force field.

Docking studies were carried out using a Monte Carlo/Simulated Annealing (SA) docking methodology, which considers all the system flexible (Affinity, SA Docking; Insight 2005, Accelrys, San Diego, CA) (Senderowitz et al. 1995) and using the Cell Multipole method for non-bond interactions (Ding et al. 1992). Although all the system (i.e., ligand, protein, and water molecules) is perturbed by Monte Carlo and simulated annealing (SA) calculations in the subsequent dynamic docking protocol, the dynamic docking procedure formally requires a reasonable starting complex structure. To increase the variance of the starting complexes (i.e., starting ligand poses), two AFB1 starting complexes were used for each receptor, for a total of four sets of docking calculations. In particular, AFB1 was positioned: i) in hVDR LBD according to the two superimpositions on 1,25(OH)_2_D_3_ (PDB ID: 1DB1) reported in Fig. 2A; ii) in hRXRα LBD according to the two superimpositions on the 9-cis retinoic acid (PDB ID: 1FBY) reported in Fig. 2B. The binding domain area was defined as a flexible subset around the ligand constituted by all residues and water molecules having at least one atom within a 10 Å radius from any given ligand atom. The atoms included in the binding domain area were left free to move during docking calculations. A restrain buffer region was introduced to separate the freely movable atoms and non-movable atoms. If the closest distance of a movable atom to bulk atoms was less than the sum of their van der Waals radii plus the 0.5 Å, that movable atom was restrained to its original position using a harmonic restrain force of 100 kcal mol^−1^ Å^−1^.

The docking protocol included a Monte Carlo based conformational search of AFB1 within the defined active site. A Monte Carlo/minimization approach was used for the random generation of a maximum of 20 acceptable complexes. During the first step, starting from the roughly docked structures, the ligand was moved by a random combination of translation, rotation, and torsional changes to sample both the conformational space of the ligand and its orientation to the protein (MxRChange = 3 Å; MxAngChange = 180°). During this step, van der Waals (vdW) and Coulombic terms were scaled to a factor of 0.1 to avoid very severe divergences in the vdW and Coulombic energies. If the energy of a complex structure resulting from the ligand's random moves was higher by the energy tolerance parameter than the energy of the last accepted structure, it was not accepted for minimization. An energy tolerance value of 10^6^ kcal/mol from the previous structure was used to ensure a wide variance of the input structures was successfully minimized. After the energy minimization step (conjugate gradient; 10,000 iterations; ε = 1), the energy test, with an energy range of 50 kcal/mol, and a structure similarity check (rms tolerance = 0.3 kcal/Å) was applied to select the 20 acceptable structures. Each subsequent structure was generated from the last accepted structure. The resulting docked structures were ranked by their conformational energy. Finally, to test the thermodynamic stability of the resulting docked complexes, these latter were subjected to a molecular dynamic simulated annealing protocol using the Cell_Multipole method for non-bond interactions and the dielectric constant of the water (ε = 80*r.) The protocol included 5 ps of a dynamic run divided into 50 stages (100 fs each), during which the system's temperature was linearly decreased from 500 to 300 K (Verlet velocity integrator; time step = 1.0 fs). In simulated annealing, the temperature was altered from an initial temperature to a final temperature in time increments. The temperature was changed by adjusting the kinetic energy of the structure (by rescaling the velocities of the atoms). Molecular dynamics calculations were performed using a constant temperature and constant volume (NVT) statistical ensemble and the direct velocity scaling as temperature control method (temp window = 10 K). In the first stage, initial velocities were randomly generated from the Boltzmann distribution, according to the desired temperature, while during the subsequent stages, initial velocities were generated from dynamics restart data. The temperature of 500 K was applied to surmount torsional barriers, thus allowing an unconstrained rearrangement of the "ligand" and the "protein" active site (initial vdW and Coulombic scale factors = 0.1). Successively temperature was linearly reduced to 300 K in 5 ps, and, concurrently, the vdW and Coulombic scale factors have been similarly increased from their initial values (0.1) to their final values (1.0). A final round of 10^5^ minimization steps (ε = 80*r) followed the last dynamics steps, and the minimized structures were saved in a trajectory file. The complexes obtained by docking studies were ranked by conformational energy values and non-bond interaction energy values (vdW and electrostatic energy contribution; Group-Based method; CUT_OFF = 100; ε = 1; Discover_3 Module of Insight2005). The complex with the best compromise among these two parameters was selected as the structure representing the most probable binding mode.

In order to allow the whole relaxation of the protein, the selected complexes (hVDR and hRXRα) were then subjected to MM energy minimization without restraints (Steepest Descent algorithm; ε = 1) until the maximum RMS derivative was less than 0.1 kcal/Å (Module Discover; Insight 2005). The protein structural quality in the resulting complex was then checked using Procheck (Laskowski et al. 1993).

Ligand-induced protein conformational changes and ligand–protein interactions of the final AFB1/hVDR and AFB1/hRxRα docked complexes were analyzed and compared to those obtained by the analysis of the experimentally determined complexes as reported in the above paragraph.

### Cell cultures and treatments

The Saos-2 human osteosarcoma cell line was cultured in DMEM (Gibco-Thermo Fisher Scientific, Inc. Waltham, MA, USA) supplemented with 10% (v/v) fetal bovine serum (Invitrogen-Thermo Fisher Scientific Waltham, Massachusetts, USA) at 37 °C in an atmosphere containing 5% CO_2_. Cells were passaged according to standard cell culture techniques. Treatments with vitamin D3 and Aflatoxin B1 were performed as follows: Saos-2 cells were plated at a density of 3 × 10^5^cells/well in 6-well plates and were exposed for 24 h with single treatments of AFB1 (0.1 μM) and vitamin D3 (0.1 μM) or different combined treatments with a fixed dose of vitamin D3 (0.1 μM) and increasing amounts AFB1 (0.05, 0.1 and 0.2 μM). A vehicle control (0.05% DMSO) was included in each experiment. Twenty-four hours after treatments, Saos-2 cells were harvested for RNA and protein analysis.

### Transient transfections and dual-luciferase reporter assays

Saos-2 cells were seeded at a density of 8 × 10^4^ cells per well onto 12-well culture dishes and transiently transfected using Lipofectamine LTX (Invitrogen, Thermo Scientific) as previously reported (Sodaro et al. 2018a, b). A reporter plasmid containing a 960 bp fragment (-960/ + 1 nt) of the human proximal VDR promoter region was cloned upstream of the luciferase reporter gene (pVDR/Luc). Each well received 490 ng of pVDR/Luc plasmid and 10 ng of a Renilla luciferase construct (pRL-SV40, Promega, Madison, USA) as an internal control. All transfection experiments were conducted in triplicate. Aflatoxin B1 and vitamin D3 treatments were applied 4 h after transfection for each experimental point. After 24 h, cells were lysed and used for the dual-luciferase assays (Dual-Luciferase® Reporter assay system, Promega) as previously described (Sarnelli et al. 2017). All relative luciferase activities were determined by calculating the ratio of the firefly and Renilla luciferase activities, and the results are shown as mean ± SEM (n = 3). For Real-time PCR, RNAs were extracted from Saos-2 cells using Qiazol reagent (Qiagen, GmbH, Hilden, Germany) according to the manufacturer's protocol. One microgram of each RNA was reverse transcribed using QuanTitect Reverse transcription Kit (Qiagen) as reported by manufacturer's protocol and subsequently used for Real-time RT-PCR procedures on a CFX Real-time System (Bio-Rad Laboratories, Hercules, CA, USA). Real-time quantitative analysis of VDR transcripts was performed using primers as previously reported, and β actin mRNA was used as endogenous control (Faniello et al. 2009). Real-time PCR reactions were run in triplicates using the CFX96 Real-Time System (Bio-Rad Laboratories), and CT values were obtained from automated threshold analysis. Data were analyzed with the CFX Manager 3.0 software (Bio-Rad Laboratories) according to the manufacturer's specifications.

### Chromatin Immunoprecipitation

Chromatin Immunoprecipitation assays were performed as described (Sodaro et al. 2018a, b). Briefly, Saos-2 cells were chemically cross-linked with 1% formaldehyde, and the reaction was stopped by adding glycine to a final concentration of 125 mM. The fixed cells were washed twice with cold phosphate-buffered saline (PBS 1X) and were lysed using a lysis buffer (5 mM PIPES; 85 mM KCl; 0,5% NP40) supplemented with a protease inhibitor cocktail (Sigma Aldrich). Nuclei were isolated and sonicated in a buffer containing 1% SDS; 10 mM EDTA; 50 mM Tris HCl pH 8.0. The resulting fragments were within the size range of 200–1,000 bp. Samples were then centrifuged at 13,000 × g for 10 min at 4 °C, and the supernatant was pre-cleared with 30 μL protein A/G PLUS-agarose beads for 2 h and incubated with 2 μg of each antibody [RXRα (D-20X) cat. no. sc-553X; RARα(C-20X) cat. no. sc-551X; VDR (C-20X) cat. no. sc-1008X; Santa Cruz Biotechnology, Dallas, TX, USA] overnight at 4 °C. Rabbit IgG antibody (sc-2027X, Santa Cruz Biotechnology) served as a negative control. Following chromatin immune-precipitation, beads were then rinsed five times with buffer A [0,1% SDS; 2 mM EDTA; 20 mM Tris HCl pH 8,0; 1% Triton X-100; 150 mM NaCl], four times with buffer B [0,1% SDS; 2 mM EDTA; 20 mM Tris HCl pH 8,0; 1% Triton X-100; 500 mM NaCl], and once with Tris–EDTA pH buffer. The bound immunocomplexes were eluted by adding 300 μL of fresh elution buffer [10 mM Tris; 1 mM EDTA pH 8.0]. Subsequently, 20 μL of 5 M NaCl was mixed with the eluted product, incubated overnight at 65 °C to reverse the cross-linking. Immunoprecipitated genomic DNA was then purified and dissolved in EB buffer (10 mM Tris; 1 mM EDTA pH 8.0) for ChIP analysis. The immunoaffinity-enriched DNA was subjected to quantitative real-time PCR analysis using SSO Advanced Universal SYBR Green Supermix by CFX96 Detection System (Bio-Rad Laboratories). The primer pairs used in the present study were as follows: VDR promoter For 5′-TCCGCACCTATAATCATCGAC-3′, VDR promoter Rev 5′-GCCACGCTGTAGCCTTAGAT- 3'; VDR enhancer S1 For 5′-CAACTGTCCCAGGCCTGAG-3′, VDR enhancer S1 Rev 5′-GGTGGGGCAACCAAGCTAA-3', HBB LCR region (used as negative control): HS2 For 5’-CCCTGTCGGGGTCAGTGCC-3', HS2 Rev 5’-CACATTCTGTCTCAGGCATCC-3'. The Ct values of specific antibodies and IgG control were normalized to the input values (∆Ct = Ct Ip_VDR/RXRaα/RARα_ or IgG-Ct_Input_). The fold enrichment was calculated by the ∆∆Ct cycle threshold method by comparing the ChIP antibody signal to the corresponding IgG negative control (Fold enrichment = ∆∆Ct = 2^-(∆Ct Ip_VDR/RXRα/RARα_-∆Ct IgG). Results are representative of two independent experiments.

### Western blot analysis

Saos-2 cells were washed with PBS and lysed in whole-cell extract buffer (50 mM Tris–HCl, pH 8; 10% glycerol; 150 mM NaCl; 1 mM EDTA pH 8; 0.1% Nonidet P-40; and 1 mM NaF) supplemented with a protease inhibitor cocktail (complete cocktail; Sigma Aldrich, St. Louis, USA). According to the manufacturer's protocol, differential nuclear and cytoplasmic protein extracts were carried out using the NE-PER Reagents Kit (Thermo Scientific, Waltham, USA). Western blot analysis was performed as previously described (Di Caprio et al. 2015). Whole-cell extracts (30 μg) and/or differential cytosolic and nuclear extracts (15 μg) were separated by 10% SDS–polyacrylamide gel electrophoresis and electroblotted onto a nitrocellulose membrane. The membranes were then blocked with 5% non-fat milk in Tris-buffered saline for 2 h and hybridized overnight at 4 °C to an anti-VDR rabbit antibody (1:500 dilution; Santa Cruz Biotechnology, Dallas, USA #sc-1008), anti-RXRα rabbit antibody (1:500 dilution; Santa Cruz Biotechnology, #sc-553X), or anti-RARα rabbit antibody (1:500 dilution; Santa Cruz Biotechnology, # sc-551X). Following washing, the membranes were incubated with peroxidase-conjugated mouse anti-rabbit IgG (sc-2357 diluted 1: 5.000; Santa Cruz Biotechnology, Dallas, USA) secondary antibodies for 1 h at room temperature. Anti-GAPDH (1:1000 dilution; Cell Signaling, Danvers, MA, USA #21,118), anti-Vinculin (1:10,000 dilution; Abcam, Cambridge, UK #129,002), and anti-Lamin B1 (1:1000 dilution; Cell Signaling, #13,435) antibodies were used to normalized respectively whole, cytosolic and nuclear extract samples. The blots were developed using the ECL Immobilon Western Chemiluminescent HRP-substrate system (Millipore, Darmstadt, Germany) according to the manufacturer's protocol, and immunoreactive bands were detected by autoradiography according to the manufacturer's instructions or by ChemiDoc XRS Image System (Bio-Rad Laboratories). Quantification of western blots bands was performed using the ImageJ software.

### Statistical analysis

All data were assessed as the mean ± standard deviation (SD) of at least three separate experiments performed in triplicate. Graphpad Prism 7 (Graphpad Software, Inc. CA, USA) was used for data analysis. Statistical differences were determined through the One-Way analysis of variance procedure followed by Dunnett's multiple comparison test, comparing results between mock control and treated cells. Differences were considered significant when *p* < 0.05 and highly significant when *p* < 0.0001. *p < 0.05, **p < 0.0001 versus mock control; #*p* < 0.05, ##*p* < 0.0001 single treatment with vitamin D3 (0.1 μM) versus combined treatment with increasing doses of AFB1.

## Supplementary Information

Below is the link to the electronic supplementary material.

Supplementary file1 (DOCX 496 KB)

## Data Availability

Data access: Data supporting Figs. 1, 2, 3, 4, 5, 6, 7, 8, 9, 10; Fig. 1SI-3SI, Tables 1 and 2; and Tables 1SI and 2SI are available on request from Prof. Caterina Fattorusso Department of Pharmacy, University of Naples Federico II Italy; email: caterina.fattorusso@unina.it. In particular, Fig. 1 was generated with ChemDraw 8.0, Figs. 2, 3, 4 and 10 as well as Fig. 1SI-3SI were generated using Discovery Studio 2017 (Dassault Systèmes BIOVIA, San Diego, 2017), modified with PowerPoint (Microsoft Office 15 version), and refined with GIMP 2.10.12. Tables 1–2 and Tables 1SI and 2SI can be accessed on request from prof. Caterina Fattorusso Department of Pharmacy, University of Naples Federico II Italy. Figures 5, 6, 7, 8, 9 and Fig. 1SI and Fig. 11 are available on request from Prof. Michela Grosso Department of Molecular Medicine and Medical Biotechnology, University of Naples Federico II Italy: email michela.grosso@unina.it.
