# Supplementary material for: A multidisciplinary approach disclosing unexplored Aflatoxin B1 roles in severe impairment of vitamin D mechanisms of action
Source: Cell Biol Toxicol. 2022 Sep 6;39(4):1275–95. doi: 10.1007/s10565-022-09752-y (PMC10425525; doi:10.1007/s10565-022-09752-y)
Supplement: Supplementary file 1 — Supplementary file1 (DOCX 496 KB) [file 10565_2022_9752_MOESM1_ESM.docx]

SUPPORTING INFORMATION

A multidisciplinary approach disclosing unexplored Aflatoxin B1 roles in severe impairment of vitamin D mechanisms of action

**Table of Contents:**

**Table 1SI**

**Table 2SI**

**Figure 1SI**

**Figure 2SI**

**Figure 3SI**

**Table 1SI.** Conformational energy difference (ΔE) and nonbond interactions of the 40 AFB1/VDR complexes produced by the docking simulation using the two starting binding modes reported in Figure 2.

| **Starting binding mode** | **Frame** | **ΔE_GM_** | **Nonbond interaction**  **(kcal/mol)** |
| --- | --- | --- | --- |
| I | 1 | 21.984 | -29.277 |
|  | 2 | 33.233 | -30.884 |
|  | 3 | 9.494 | -31.922 |
|  | 4^a^ | 0.000 | -31.365 |
|  | 5 | 21.607 | -32.346 |
|  | 6 | 24.594 | -33.489 |
|  | 7 | 23.053 | -33.036 |
|  | 8 | 8.712 | -32.079 |
|  | 9 | 21.992 | -28.996 |
|  | 10 | 43.820 | -29.498 |
|  | 11 | 3.502 | -31.947 |
|  | 12 | 13.623 | -32.803 |
|  | 13 | 52.597 | -28.561 |
|  | 14 | 24.185 | -29.573 |
|  | 15 | 48.890 | -32.438 |
|  | 16 | 15.792 | -32.983 |
|  | 17 | 21.695 | -26.285 |
|  | 18 | 34.951 | -31.614 |
|  | 19 | 46.975 | -30.442 |
|  | 20 | 44.490 | -37.328 |
| II | 1 | 10.898 | -32.250 |
|  | 2 | 27.927 | -30.588 |
|  | 3 | 2.480 | -31.934 |
|  | 4 | 69.110 | -30.898 |
|  | 5 | 46.930 | -37.268 |
|  | 6 | 57.470 | -32.706 |
|  | 7 | 19.120 | -35.134 |
|  | 8 | 37.262 | -29.930 |
|  | 9 | 4.979 | -32.536 |
|  | 10 | 19.466 | -31.785 |
|  | 11 | 8.992 | -35.881 |
|  | 12 | 8.839 | -30.047 |
|  | 13 | 23.334 | -32.174 |
|  | 14 | 52.091 | -33.814 |
|  | 15 | 38.282 | -35.571 |
|  | 16 | 22.588 | -36.991 |
|  | 17 | 19.345 | -28.585 |
|  | 18 | 38.281 | -31.355 |
|  | 19 | 1.758 | -33.429 |
|  | 20 | 20.636 | -29.383 |

^a^ Selected AFB1/VDR docked complex.

**Table 2SI.** Conformational energy difference (ΔE) and nonbond interactions of the 40 AFB1/RXRα complexes produced by the docking simulation using the two starting binding modes reported in Figure 2.

| **Starting binding mode** | **Frame** | **ΔE_GM_** | **Nonbond interaction**  **(kcal/mol)** |
| --- | --- | --- | --- |
| I | 1 | 65.090 | -30.559 |
|  | 2 | 6.342 | -34.270 |
|  | 3 | 35.600 | -32.879 |
|  | 4 | 71.295 | -28.628 |
|  | 5 | 35.315 | -30.962 |
|  | 6 | 42.266 | -24.151 |
|  | 7 | 43.859 | -23.884 |
|  | 8 | 67.618 | -27.884 |
|  | 9 | 48.841 | -22.976 |
|  | 10^a^ | 0.000 | -32.269 |
|  | 11 | 42.845 | -36.518 |
|  | 12 | 28.516 | -29.281 |
|  | 13 | 29.650 | -35.038 |
|  | 14 | 39.164 | -23.849 |
|  | 15 | 59.908 | -32.318 |
|  | 16 | 47.251 | -35.323 |
|  | 17 | 51.515 | -41.728 |
|  | 18 | 25.304 | -34.106 |
|  | 19 | 70.721 | -32.322 |
|  | 20 | 49.445 | -32.425 |
| II | 1 | 43.261 | -27.890 |
|  | 2 | 52.867 | -29.078 |
|  | 3 | 15.761 | -32.906 |
|  | 4 | 35.061 | -36.691 |
|  | 5 | 45.976 | -30.106 |
|  | 6 | 70.773 | -33.329 |
|  | 7 | 49.036 | -31.715 |
|  | 8 | 28.689 | -27.742 |
|  | 9 | 39.036 | -26.242 |
|  | 10 | 49.916 | -34.322 |
|  | 11 | 43.995 | -35.554 |
|  | 12 | 59.120 | -42.432 |
|  | 13 | 39.374 | -25.181 |
|  | 14 | 46.068 | -26.815 |
|  | 15 | 70.018 | -30.621 |
|  | 16 | 54.704 | -30.219 |
|  | 17 | 70.888 | -31.673 |
|  | 18 | 33.156 | -32.291 |
|  | 19 | 99.522 | -26.720 |
|  | 20 | 42.406 | -38.407 |

^a^ Selected AFB1/RXRα docked complex.


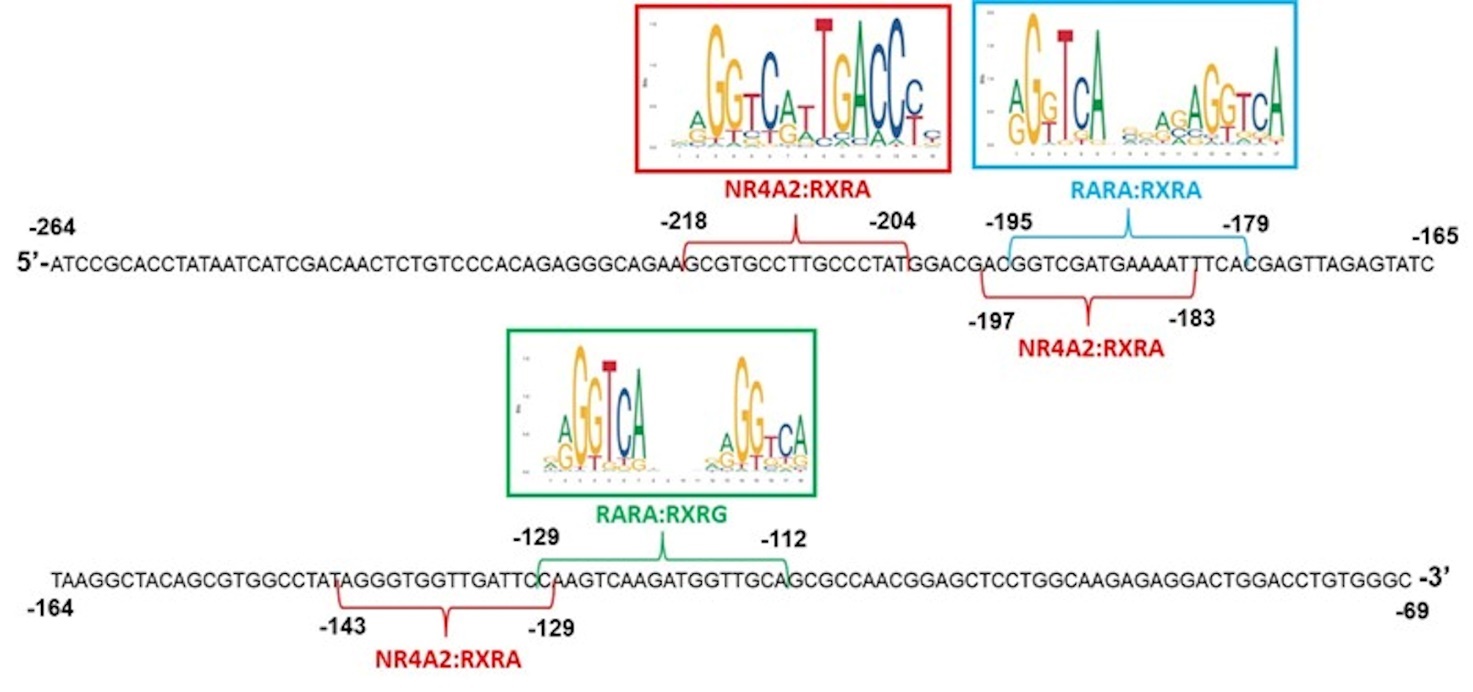


**Figure 1SI.** Predicted RXR-based heterodimer responsive elements on the fragment from -264 to -69 bp of the VDR promoter using the database of transcription factor binding profiles JASPAR and a profile score threshold value ≥ 75. RXRA: Nuclear receptor RXRα; RARA: Nuclear receptor RARα; RXRG: Nuclear receptor RXRγ; NR4A2: Nuclear receptor subfamily 4 group A member 2.


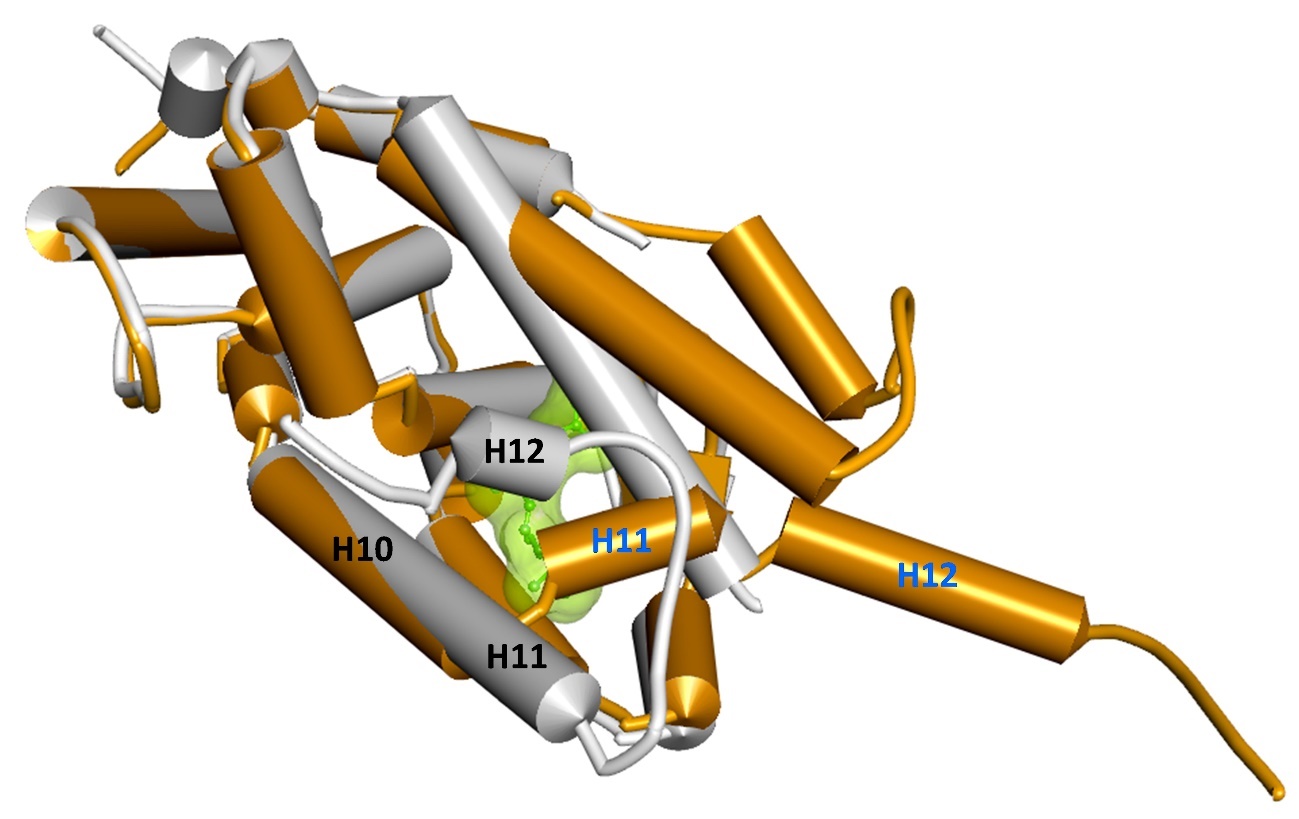


**Figure 2SI.** Superimposition by the Cα atoms of the X-ray structure of the apo structure of the LBD of RXRα (orange; PDB ID: 6HN6) on the X-ray structure of the LBD of RXRα in complex with the 9-cis retinoic acid (ligand: green; protein: gray) (PDB ID: 1FBY). The protein structures are displayed as follows: helical structures as wide cylinders, beta-sheets as arrows, and coil and turn regions as tubes. H10, H11 and H12 helices involved in the transactivation are labeled. The ligand is displayed in ball&stick and its solvent accessible surface is showed.

**
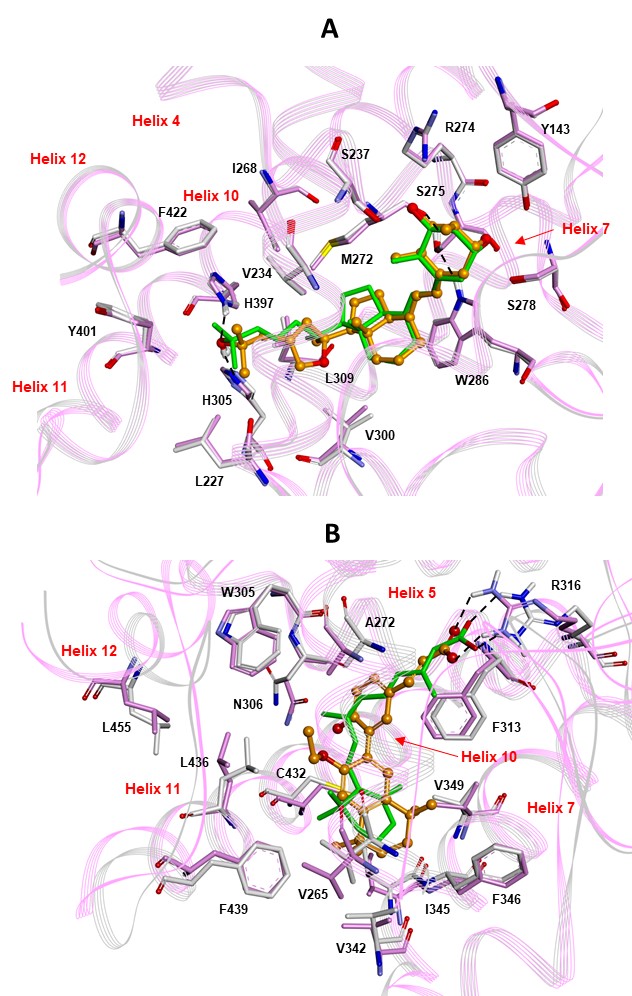
**

**Figure 3SI.** A: Superimposition by the Cα atoms of the X-ray structures of hVDR LBD in complex with: i) the synthetic agonist 2alpha-methyl-AMCR277B (agonist: orange; VDR: pink) (PDB ID: 3A40), ii) the endogenous agonist 1,25(OH)_2_D_3_ (ligand: green; VDR: gray) (PDB ID: 1DB1). B: Superimposition by the Cα atoms of the X-ray structures of hRXRα LBD in complex with: i) the synthetic agonist compound 3-(2'-ethoxy)-tetrahydronaphtyl cinnamic acid (agonist: orange; RXRα: pink) (PDB ID: 2P1U), ii) the endogenous agonist 9-cis retinoic acid (ligand: green; protein: gray) (PDB ID: 1FBY). Heteroatoms are colored by atom type (O = red; N = blue; S = yellow). Black dashed lines highlight hydrogen bonds. Hydrogens are omitted for clarity except those involved in hydrogen bond interactions.
